# ATP‐Regulated Formation of Transient Peptide Amphiphiles Superstructures

**DOI:** 10.1002/smll.202410850

**Published:** 2025-02-25

**Authors:** David Cappelletti, Federico Lancia, Andrea Basagni, Luka Đorđević

**Affiliations:** ^1^ Department of Chemical Sciences University of Padova Via Marzolo 1 Padova 35131 Italy; ^2^ Via Senorbi 28 Roma 00148 Italy

**Keywords:** ATP, nanostructures, peptide amphiphiles, supramolecular chemistry, systems chemistry

## Abstract

Self‐assembly of biotic systems serves as inspiration for the preparation of synthetic supramolecular assemblies to mimic the structural, temporal, and functional aspects of living systems. Despite peptide amphiphiles (PAs) being widely studied in the context of biomimetic and bioactive functional nanomaterials, very little is currently known about the reversible and spatiotemporal control of their hierarchical self‐assemblies. Here, it is shown that PA‐based supramolecular nanofibers can transiently form superstructures, through binding with oppositely charged adenosine triphosphate (ATP), leading to charge screening and stabilization of bundled nanofibers. Enzymatic hydrolysis of ATP to adenosine monophosphate and phosphates causes the disassembly of the superstructures and recovery of individual nanofibers. The lifetime of superstructures can be controlled by adjusting the concentration of either ATP or enzyme. The role that the formation of bundled PA nanofibers has on chemical reactivity and catalysis is also evaluated. It is observed that superstructuration is responsible for downregulation in the PA activity, which can then be recovered by gradual disassembly of the bundles. These results demonstrate the potential of reversible and controlled hierarchical self‐assembly to modulate the reactivity and catalysis of peptide nanostructures.

## Graphical abstract

This work reports the formation of peptide amphiphile bundled superstructures through ATP‐driven charge screening. The transient nature of these hierarchical self‐assembled nanostructures is regulated by enzymatic ATP hydrolysis. Using this approach also controls the chemical reactivity and catalytic function of peptide nanostructures.

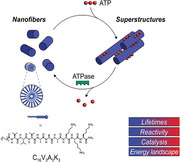

## 1 Introduction

Living systems operate out‐of‐equilibrium, through chemical reaction networks, to accomplish spatiotemporal regulation of various important functions such as cell division, motility, and signal transduction.^[^ ^1^, ^2^, ^3^ ^]^ Self‐assembly of biological systems under dissipative conditions acts as inspiration for the preparation of supramolecular systems that mimic structural, temporal, and functional aspects of natural assemblies.^[^ ^4^, ^5^, ^6^, ^7^, ^8^ ^]^ Given that biological systems sustain out‐of‐equilibrium conditions with energy continuously supplied by carriers such as adenosine triphosphate (ATP) (and guanosine triphosphate), it is immediately apparent why ATP has been used to control the spatiotemporal assembly of abiotic molecules and materials. Since seminal reports in which the addition of ATP and its hydrolysis has been used to control the transient generation of fluorescence signals^[^ ^9^ ^]^ and surfactant micelles,^[^ ^10^ ^]^ several other systems have been developed. Examples include supramolecular polymerization of chromophores,^[^ ^11^, ^12^, ^13^ ^]^ co‐assembly of colloidal particles,^[^ ^14^ ^]^ and control over conformational switching between different nanostructures,^[^ ^15^, ^16^, ^17^, ^18^, ^19^ ^]^ to name a few. Besides chemical reaction networks based on ATP, other abiotic alternatives have also been successfully developed. Transient morphological changes of supramolecular systems have also been accomplished through redox reactions,^[^ ^20^, ^21^, ^22^, ^23^ ^]^ carbodiimide‐fueled reactions,^[^ ^24^, ^25^, ^26^, ^27^ ^]^ dynamic imine bonds formation,^[^ ^28^, ^29^, ^30^ ^]^ methylation,^[^ ^31^, ^32^, ^33^ ^]^ as well as pH‐switching.^[^ ^34^, ^35^, ^36^ ^]^

To develop bio‐inspired supramolecular systems, besides employing biological energy carriers like ATP, using (bio)molecules or their synthetic derivates as monomers is another intense area of research. In this context, peptides and their amphiphilic derivates have been exploited to prepare self‐assembled nanostructures that show promising applications, especially in tissue regeneration, targeted therapies, and drug delivery.^[^ ^37^, ^38^, ^39^, ^40^, ^41^, ^42^, ^43^, ^44^ ^]^ Specifically, peptide amphiphiles (PAs) are a class of molecules that consists of an oligopeptide segment conjugated to a hydrophobic domain, commonly in the form of lipid tails.^[^ ^45^, ^46^ ^]^ These molecules tend to self‐assemble into 1D nanostructures, which can then form hydrogel networks. While many studies have elucidated how their sequence affects the self‐assembly,^[^ ^47^, ^48^, ^49^ ^]^ and the conditions in which they self‐assemble (PA concentration, pH, temperature, salt, etc.),^[^ ^50^, ^51^, ^52^, ^53^, ^54^, ^55^, ^56^, ^57^, ^58^ ^]^ much less work has been done in studying their reversible self‐assembly. In a pioneering study, it was shown that PA derivates could form superstructures made of bundled nanofibers, which could then be disassembled back into nanofibers by adding a chemical trigger.^[^ ^59^ ^]^ The resulting hydrogels were used as extracellular matrix mimics and, depending on the presence of superstructures, the phenotype of the cultured cells was observed to change. However, switching between these two states was not repeated and was dependent on the addition of external chemical triggers, thus it could not be controlled in a spatiotemporal and repeated manner. Similar observations were reported for bundled PA nanofibers obtained through host‐guest interactions.^[^ ^60^, ^61^, ^62^ ^]^ Other methods reported for the transient self‐assembly of amphiphilic peptides are through chemical energy carriers (such as carbodiimides)^[^ ^63^, ^64^, ^65^ ^]^ or enzyme‐catalyzed pH jumps^[^ ^66^, ^67^ ^]^ or light.^[^ ^68^, ^69^ ^]^ However, these usually do not allow reversible and transient superstructuration. This remains an open research goal, which we believe could be achieved by applying a systems chemistry approach to PAs, thus allowing us to prepare transient hierarchical self‐assembled nanostructures. Previous works, relying on short peptide sequences, have revealed that such an approach could be successful, with the transient self‐assembly being controlled either by competitive catalytic transacylation,^[^ ^70^, ^71^ ^]^ reversible covalent fuel linkage,^[^ ^72^, ^73^, ^74^, ^75^ ^]^ carbodiimides,^[^ ^76^, ^77^ ^]^ or activated carboxylic acids.^[^ ^78^ ^]^ Feasibility is also indicated through recent reports in which an enzyme‐modulated adenosine triphosphate system has been applied to positively charged oligopeptides but was used to study transient formation of condensates through liquid‐liquid phase separation.^[^ ^79^, ^80^, ^81^ ^]^

**Figure 1:**
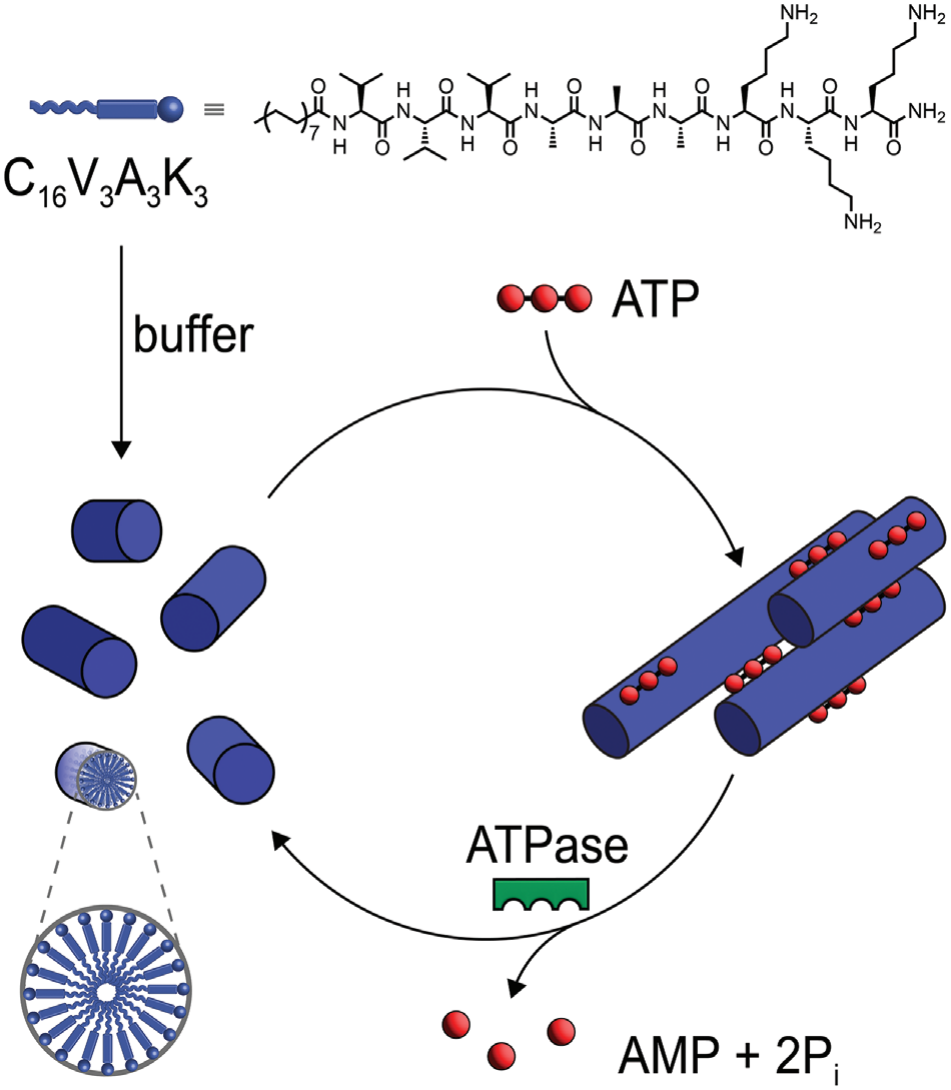
Schematic representation of the peptide amphiphile‐ATP system studied in this work. The PA (with a chemical structure of the C_16_V_3_A_3_K_3_, top) in buffer solution self‐assembles into short nanofibers. The bundling of these nanofibers is regulated by the addition of ATP, while their disassembly is achieved in the presence of an enzyme for ATP hydrolysis (ATPase).

In this work, we have developed a system that shows transient superstructure formation making use of peptide amphiphile (PA)‐based supramolecular nanostructures in solution (**Figure** **1**). To achieve this, the positively charged nanofibers (C_16_V_3_A_3_K_3_) were charge screened in the presence of ATP that carries three negatively charged phosphate groups, thus leading to the formation of bundles. The superstructures were found to reversibly disassemble in solution in the presence of an ATPase enzyme, which hydrolyzes ATP to adenosine monophosphate (AMP) and phosphates (P_i_). The resulting transient bundles were characterized by spectroscopic and microscopic techniques, and the effect of superstructure formation was assessed in chemical reactivity and catalysis experiments.

## 2 Result

### 2.1 System Design and Morphological Studies

**Figure 2:**
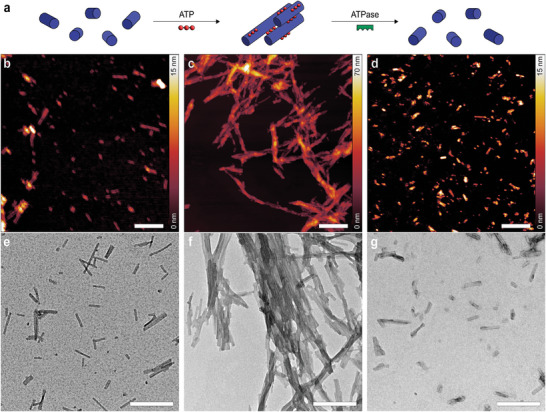
a) Schematic representation of the peptide amphiphile‐ATP system. b,d) Micrographs of the C_16_V_3_A_3_K_3_ self‐assembled short nanofibers before the addition of ATP, c,f) after the addition of ATP, and d,g) finally after the addition of ATPase. AFM (top row, scale bars are 500 nm) and TEM (bottom row, scale bars are 250 nm) images were acquired either in the absence or presence of ATP (60 µm), or after the enzyme action (60 µm ATP and 10 U mL^−1^ potato apyrase in solution). Experimental conditions: C_16_V_3_A_3_K_3_ (100 µm), HEPES (10 mm, 7.2 pH), CaCl_2_ (1.0 mm).

We sought to employ the C_16_V_3_A_3_K_3_ peptide amphiphile, with a sequence V_3_A_3_K_3_ conjugated to a 16‐carbon alkyl chain at the N‐terminus (Figure 1, top). This PA consists of a palmitic acid tail conjugated to three valine and three alanine residues, to promote intermolecular β‐sheet hydrogen bonding, and finally three lysine residues, to impart aqueous solubility, amphiphilicity, and chemical reactivity to the monomers. It was previously reported that C_16_V_3_A_3_K_3_ can self‐assemble in water into different aggregate morphologies.^[^ ^53^ ^]^ Due to the presence of charged lysine residues in the peptide, the self‐assembly is sensitive to ionic strength: at low ionic strength, the PA self‐assembles into polydisperse fibers with a predominant random coil, whereas at high ionic strength, the fibers show a β‐sheet secondary structure.^[^ ^53^ ^]^ We hypothesized that ATP could electrostatically interact with the positively charged PA polydisperse nanofibers, causing charge screening and inducing the formation of superstructures (**Figure** **2**a). Then, in the presence of an ATPase enzyme, which hydrolyzes ATP into adenosine monophosphate and two equivalents of inorganic phosphate,^[^ ^82^ ^]^ we expected the disassembling of the superstructures (Figure 2a), due to decreased electrostatic interaction and lower charge screening.

The morphology of freshly dissolved C_16_V_3_A_3_K_3_ PA in an aqueous buffer solution was studied by atomic force microscopy (AFM) and transmission electron microscopy (TEM). A typical solution consisted of C_16_V_3_A_3_K_3_ (100 µm) in HEPES buffer (10 mm, at pH 7.2 to avoid changes in pH during ATP addition and hydrolysis) and CaCl_2_ (1.0 mm, ATPase enzyme co‐factor).^[^ ^9^, ^83^ ^]^ AFM (Figure 2b) and TEM (Figure 2e) experiments reveal the presence of polydisperse short nanofibers of 12 ± 2 nm in height and 63 ± 31 nm in length, respectively. Furthermore, circular dichroism (CD) confirms the β‐sheet secondary structure of these nanofibers (Figure S2, Supporting Information). These observations are consistent with the morphology reported for freshly dissolved C_16_V_3_A_3_K_3_ in high ionic strength conditions.^[^ ^53^ ^]^

The microscopic characterization was also performed after the addition of ATP (60 µm) to the PA (100 µm) nanofibers solution. AFM and TEM (Figure 2c,f; Figure S3, Supporting Information) show the formation of large bundles after adding ATP to the aqueous PA buffer solution. Specifically, hierarchical nanostructures with a height of 40 ± 13 nm are observed by AFM, ≈30 nm higher than individualized nanofibers when ATP is not present. The length of bundled nanofibers is 181 ± 74 nm, as shown by TEM, evidencing an increased length of the nanofibers upon the addition of ATP. Scanning electron microscopy (SEM) was also used to examine the structure of the PA‐ATP system and show the presence of a network of bundled fibers (Figure S4, Supporting Information). The observed morphology is qualitatively similar to those of previously reported host‐guest PA superstructures.^[^ ^60^, ^61^, ^62^ ^]^ Dynamic light scattering (DLS) also shows that supramolecular bundling occurs after ATP addition since a large increase in the hydrodynamic diameter is noticeable (Figure S5, Supporting Information).

Finally, we added ATP (60 µm) to a solution containing PA and ATPase (potato apyrase, 10 U mL^−1^) to probe if the system is reversible and if the hydrolysis of ATP (over 60 min) would lead the bundles to disassemble (Figure 2d,g). Both AFM and TEM images show similarities to the individualized nanostructures observed before ATP addition (Figure 2b,e), thus proving the reversibility of the system. Specifically, polydisperse short nanofibers of ≈12 ± 3 nm in height and 100 ± 39 nm in length are found by AFM and TEM imaging, respectively. DLS also confirmed the disassembling of the bundles, as the observed hydrodynamic diameter decreases to a value similar to that of the nanofibers without ATP present (Figure S5, Supporting Information). We therefore conclude that the formation of superstructures can be regulated by the addition and subsequent hydrolysis of ATP.

**Figure 3:**
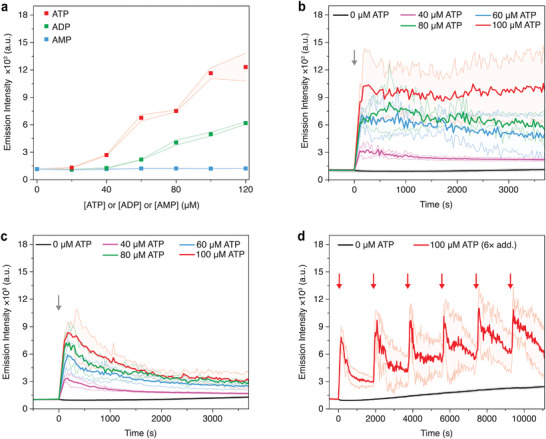
a) Fluorescence intensity of ThT as a function of concentration of ATP, ADP, and AMP. Different amounts of ATP, ADP, and AMP (0–120 µm) were added to aqueous buffer solutions containing only PA and ThT. b) Kinetic fluorescence experiments where different amounts of ATP were added at 0 s to solutions in the absence and c) presence of potato apyrase (0.050 U mL^−1^). d) ATP (100 µm) was added six times (first addition at 0 s, cycles = 30 min) to solutions containing potato apyrase (0.10 U mL^−1^). Experimental conditions: C_16_V_3_A_3_K_3_ (100 µm), HEPES (10 mm, 7.2 pH), CaCl_2_ (1.0 mm), 30 °C, ThT (10 µm, *λ*_ex._ = 440 nm/*λ*_em._ = 483 nm). All the error bars denote standard deviation based on quadruplicate measurements.

The hierarchical self‐assembly of C_16_V_3_A_3_K_3_ in the presence of ATP (and ATPase) can be followed by optical density, but also through fluorescence emission by using thioflavin T (ThT) as probe known for its binding to amyloids (Figure S6, Supporting Information).^[^ ^80^, ^81^, ^84^ ^]^ We therefore expected that monitoring the ThT fluorescence could be used to track the reversible fibrillation and hierarchical self‐assembly. Control experiments show that the addition of ATP, when compared to additions at the same concentration of adenosine diphosphate (ADP) and monophosphate, results in higher ThT emission (483 nm). The higher emission upon the presence of ATP indicates a higher affinity of the positively charged nanofibers to interact specifically with ATP (**Figure** **3**a). As expected, a triple negatively charged molecule like ATP, compared to the same concentration of double or single negatively charged adenosine phosphates, will electrostatically interact better with the positively charged C_16_V_3_A_3_K_3_. In this regard, we performed *ζ*‐potential measurements at the same concentration of adenosine phosphates (60 µm). The *ζ*‐potential of PA nanofibers (40.5 ± 1.6 mV) decreases after the addition of ATP (27.7 ± 0.8 mV), resulting in a higher charge screening of the positively charged PA when compared with the addition of ADP and AMP (31.6 ± 0.5 mV and 36.8 ± 0.8 mV, respectively) (Figure S7, Supporting Information). We then moved to compare the same amount of negatively charged phosphate groups (e.g., 1.0 equivalent of ATP with 1.5 eq. of ADP and 3.0 eq. of AMP) and observe a higher ThT emission for the PA‐ATP solution (e.g., see points of 40 µm ATP, 60 µm ADP, and 120 µm AMP in Figure 3a), even though the surface charge remains constant from *ζ*‐potential experiments (Figure S7, Supporting Information). These experiments demonstrate the importance of multivalent interactions,^[^ ^85^, ^86^ ^]^ in which the multiple positive charges on the surface lysines of the PA nanofibers have a higher relative affinity for ATP, in which the negatively charged phosphates are covalently linked together and in close proximity. Indeed, when multivalent interactions between ATP and PA occur, better charge screening of the positively charged nanofibers occurs, followed by fibrillation and superstructure formation. When three equivalents of AMP interact with the peptide, the phosphate groups are not covalently linked as in the ATP case and, consequently, the charge screening is much less effective.

We then performed kinetic fluorescence experiments for the PA‐ATP system by following the ThT fluorescence emission. At first, the bundling of PA under variable concentrations of ATP was studied (Figure 3b), without the presence of the ATPase enzyme. The fluorescence intensity of ThT rapidly increases following the addition of ATP to solutions containing PA (100 µm) and then remains almost constant. When higher ATP concentrations are employed we observe increased fluorescence, which is expected in higher charge screening conditions and, consequently, more supramolecular bundling. It is noteworthy that fluorescence intensity also increases at lower concentrations of ATP (40 µm), indicating that the super‐structuration of PA is rather sensitive to the triple‐negatively charged molecule.

We then performed extensive kinetic fluorescence experiments to study the superstructure lifetimes and systematically changed the concentration of each component in the PA‐ATP‐ATPase system. When ATPase (potato apyrase, 0.050 U mL^−1^) was present in the PA buffer solution (100 µm), upon adding different concentrations of ATP (40–100 µm), emission intensity traces gradually decay (Figure 3c). Consistent with the microscopic evidence presented above, the emission decay observed in Figure 3c indicates that the superstructures are disassembling and that the recovery of short nanofibers can be followed by emission kinetics. Following this, we proceeded to study the addition of different amounts of ATP (100, 80, or 60 µm) to PA solutions (100 µm) that contain different enzyme concentrations (0.10, 0.050, and 0.025 U mL^−1^). Kinetic fluorescence experiments show a greater increase in emission intensity and lower decay over time after ATP addition at low ATPase concentrations (Figure S8, Supporting Information). We also changed the PA concentration in solution when the amount of enzyme and ATP were constant (0.050 U mL^−1^ and 80 µm, respectively), and observed superstructures of slightly longer lifetimes at higher PA concentrations (Figure S9, Supporting Information).

The reversibility and repeatability of the system were then demonstrated by multiple and consecutive ATP additions (performing up to six cycles with the same sample) to a solution containing PA (100 µm) and enzyme (0.10 U mL^−1^). The emission intensity signal rapidly increases each time ATP is added and then rapidly decreases, due to superstructure disassembly through enzymatic hydrolysis of ATP (Figure 3d). We note, however, that the emission intensities are slightly higher at the end of each cycle, presumably due to the accumulation of ATP hydrolysis waste products. Indeed, control experiments in the presence of AMP and two P_i_ showed higher ThT emission intensities when compared to only AMP (Figure S10, Supporting Information). This suggests that the progressive accumulation of waste could lead to incomplete debundling of PA superstructures.

### 2.2 Regulation of PA Chemical Reactivity and Catalysis

**Figure 4:**
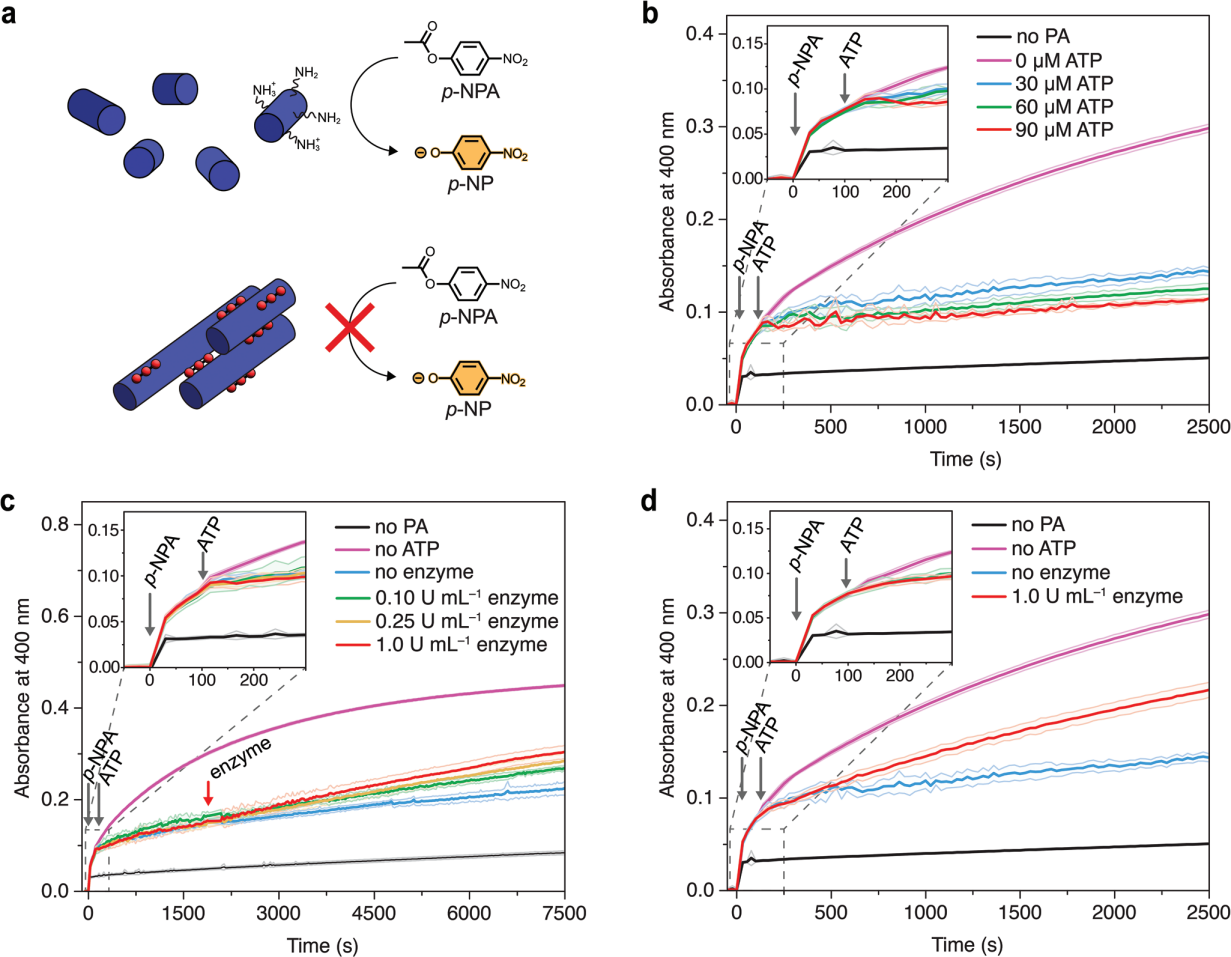
Regulation of PA chemical reactivity followed by kinetic UV–vis experiments where *p*‐NPA (500 µm) was added at 0 s and ATP after 100 s. a) Deacetylation reaction of *p*‐NPA either in the presence of PA or PA‐ATP. b) Additions of different amounts of ATP in the absence of ATPase. c) ATP addition (30 µm) followed by additions of various enzyme amounts after 1900 s. d) ATP (30 µm) added in the presence of ATPase (1.0 U mL^−1^). Experimental conditions: C_16_V_3_A_3_K_3_ (60 µm), HEPES (10 mm, 7.2 pH), CaCl_2_ (1.0 mm), 5% DMF to dissolve *p*‐NPA, T = 30 °C, *λ*_abs._ = 400 nm. All the error bars denote standard deviation based on quadruplicate measurements.

The next step was to implement a functional property to our ATP‐regulated transient system. In this regard, we sought to explore the chemical reactivity of lysine residues of C_16_V_3_A_3_K_3_ nanofibers. ATP‐templated superstructure formation is expected to alter the chemical environment around the lysine residues and, as a result, their reactivity is also expected to change. Indeed, several studies reported that the supramolecular self‐assembly often endows the system with new or altered chemical reactivities.^[^ ^72^, ^87^, ^88^ ^]^ To verify that our system may allow the reactivity to be controlled over time, we studied the deacetylation reaction of a model substrate (*p*‐nitrophenylacetate, *p*‐NPA) in aqueous buffer solutions. The progress of the reaction was monitored over time by UV–vis spectroscopy, as the *p*‐nitrophenolate deacetylation product (*p*‐NP, *λ*_abs._ = 400 nm) is obtained together with acetylated lysines of PA (**Figure** **4**a).^[^ ^89^, ^90^ ^]^

First, we explored the reactivity of lysine residues in the presence of short polydisperse nanofibers, obtained after freshly dissolving C_16_V_3_A_3_K_3_ in buffer solution, without any ATP in solution. As expected, we observe that increasing PA concentration produces more deacetylated products (Figure S11, Supporting Information). The deacetylation reaction of *p*‐NPA is promoted by deprotonated lysine residues in the PA nanofibers. The self‐assembly process of peptide amphiphiles generally involves changes to the local p*K*_a_ of amino acids, which differ from that in bulk solution.^[^ ^91^ ^]^ For instance, terminal glutamic acids in PAs are only partially charged (i.e., deprotonated) at the surface of self‐assembled nanofibers.^[^ ^92^ ^]^ Similarly, it is expected for lysine‐containing PAs that not all of them are charged and should thus be available for deacetylation of the *p*‐NPA substrate. Indeed, liquid chromatography‐mass spectrometry analysis of post‐reaction mixtures confirmed the presence of acetylated PA molecules (Figure S12, Supporting Information).

Then, we moved to study the reactivity in the presence of PA superstructures, obtained by adding various ATP concentrations to solutions containing *p*‐NPA (500 µm) and PA (60 µm), without ATPase present. In this case, for all traces, a rapid increase in 400 nm absorbance (after *p*‐NPA addition) is followed by a downregulation of the deacetylation reaction upon ATP addition, since the production of *p*‐NP almost plateaus (Figure 4b). It is apparent that, under ATP‐templating conditions, lysine residues on PA fibers are no longer available for *p*‐NPA deacetylation. This is presumably due to the bundle aggregate morphology and the ATP‐charge screening that hinders access to the lysine residues. We also quantified the extent of inhibition caused by the different concentrations of ATP added (30, 60, and 90 µm in Figure 4b). As expected, the initial rate of deacetylation for a PA solution (4.04 × 10^−8^ M s^−1^ without ATP) decreases after the addition of 30, 60, and 90 µm ATP (1.51 × 10^−8^ M s^−1^, 2.60 × 10^−9^ M s^−1^, 1.93 × 10^−9^ M s^−1^, respectively) (Figure S13, Supporting Information).

When ATPase was added to a mixture of already prepared PA‐ATP superstructures, the reactivity toward deacetylation of *p*‐PNA can be recovered (Figure 4c). Kinetic traces show that the absorbance of the deacetylated product starts to increase again after enzyme addition, which causes the disassembly of the bundles. Accordingly, the addition of higher amounts of ATPase leads to increased recovery of reactivity. However, we also observed that the reactivity of lysine residues is not fully recovered to that of PA nanofibers (without ATP), possibly due to smaller bundles still present given that the enzyme was added after superstructure formation. Overall, these experiments show that the reactivity of lysines on PAs toward the deacetylation reaction goes from a high reactivity state (*p*‐NPA addition to PA) to a low state (addition of ATP to *p*‐NPA and PA mixture), and then to a high activity state again (addition of ATPase to ATP, *p*‐NPA, and PA mixture).

At this point, we wondered whether the transient downregulation of PA reactivity was possible if the enzyme was already present before the ATP addition, instead of being added after superstructure formation (as reported in Figure 4c). Therefore, we studied the addition of ATP (30 µm) to solutions containing ATPase (1.0 U mL^−1^) and PA (60 µm) (Figure 4d). We expected an inhibition period of the deacetylation reaction, followed by an absorbance increase as a result of ATP enzymatic hydrolysis. A similar observation was previously reported in a nanoparticle‐based ATP‐driven transient system in which the catalytic activity toward the transphosphorylation of 2‐hydroxypropyl‐4‐nitrophenylphosphate was downregulated through the consumption of ATP.^[^ ^9^ ^]^ We observe a short inhibition time after ATP addition, in which both the sample with enzyme (Figure 4d, purple trace) and the sample without enzyme (Figure 4d, light blue trace) show similar absorbance traces. Subsequently, after a period of ≈5 min, the *p*‐NP production starts to increase again for the sample with enzyme, confirming the transient downregulation of the PA bundles system (Figure 4d). The addition of varying ATP concentrations was also explored, and the kinetic UV–vis experiments show again transient downregulation of lysine reactivity (Figure S14, Supporting Information).

In addition to the deacetylation reaction discussed above, we sought to study another model reaction to prove whether the (down)regulation of PA reactivity may be a general observation and could be extended to other chemical reactions. We selected the retro‐aldol reaction of methodol, which could be catalyzed by the lysine residues^[^ ^88^, ^93^, ^94^ ^]^ on PA nanofibers. The catalytic activity of PA superstructures was found temporarily downregulated also in this case, showing similar trends to the system based on the deacetylation reaction of *p*‐NPA (Figure S15, Supporting Information).

### 2.3 Energy Landscape of Self‐Assembled PAs and ATP

**Figure 5:**
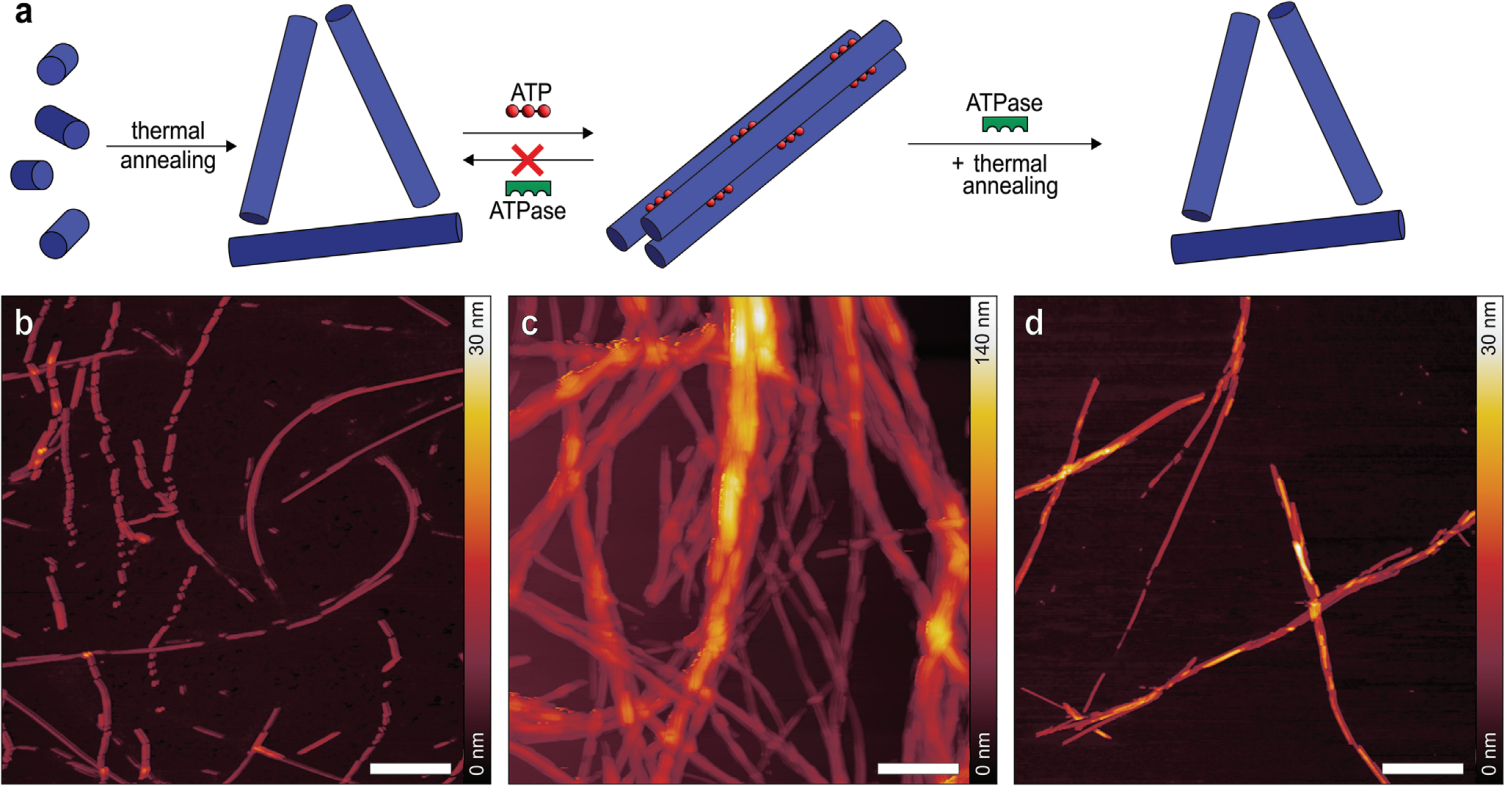
a) Schematic representation of the annealed PA‐ATP system. b) Micrograph of the annealed PA nanofibers before the addition of ATP, c) after the addition of ATP, d) and finally after the addition of ATPase. AFM images (scale bar 500 nm) were acquired either in the absence or presence of ATP (60 µm), or after the enzyme action (60 µm ATP and 10 U mL^−1^ potato apyrase in solution). Experimental conditions: C_16_V_3_A_3_K_3_ (100 µm), HEPES (10 mm, 7.2 pH), CaCl_2_ (1.0 mm).

The PA studied here (C_16_V_3_A_3_K_3_) was previously reported to form, within the same energy landscape, polydisperse fibers when freshly dissolved and long fibers when thermally annealed (thermodynamic product) under high ionic strength conditions.^[^ ^53^ ^]^ The thermal energy provided under these conditions enables nucleation and growth of long fibers with a β‐sheet secondary structure. We therefore wondered if the thermodynamically favored PA long nanofibers would behave similarly to the polydisperse freshly prepared nanofibers that we discussed above (**Figure** **5**a).

We analyzed the morphology of the PA self‐assembled system after thermal annealing (heating to 80 °C for 30 min, followed by slow cooling overnight) by atomic force microscopy. The annealed PA sample in buffer solution is made of long nanofibers with a similar height (10 ± 3 nm) and a higher β‐sheet content when compared to freshly dissolved PA nanofibers (Figure 5b; Figure S2, Supporting Information). After the addition of ATP (60 µm) to this sample, the bundling of annealed PA nanofibers is apparent. The resulting superstructures are ≈60 nm higher (68 ± 25 nm) than the annealed nanofibers without ATP present (Figure 5c). Therefore, charge screening of thermodynamically favored long nanofibers also leads to the formation of PA hierarchical self‐assembly, similar to the freshly prepared polydisperse short nanofibers.

The superstructures resulting from thermally annealed nanofibers, however, did not revert to their initial morphology when ATPase was present (10 U mL^−1^) (Figure S16, Supporting Information). Such observation was also reported by Prins and co‐workers for ATP‐templated metallo‐amphiphile assemblies.^[^ ^95^ ^]^ Their system could not return to its original state because ATP was buried inside large aggregates and not accessible to the enzyme. In our case, we observed that the thermodynamically favored nanofibers form more aggregates and bigger superstructures, compared to the ones obtained from freshly prepared solutions (compare Figures 2c and 5c). Consequently, we expect that larger aggregates prevent most of the ATP from being accessible toward enzymatic hydrolysis, preventing the disassembling of the superstructures. To support our hypothesis, we acquired scanning electron microscopy with energy‐dispersive X‐ray spectroscopy (SEM‐EDX) of PA‐ATP samples with and without the ATPase. After drop‐casting and washing, the content of phosphorous (from ATP) was found to be similar in both samples (Figure S17, Supporting Information), suggesting that ATP is likely buried within the superstructures and mostly inaccessible to the enzyme.

To re‐convert these superstructures into the initial long nanofibers, a re‐annealing process (80 °C for 30 min, then slow cooling overnight) of the solution after the ATPase addition was necessary (Figure 5d). Therefore, thermal energy was essential to make the system reversible since it likely causes the gradual loss of the superstructures and makes the ATP available for enzymatic hydrolysis. Instead of heating, we also attempted to sonicate the superstructures, which led to breaking apart not only of the bundles but also the individual long nanofibers into polydisperse short nanostructures (Figure S18, Supporting Information). Therefore, it is possible to go back to the state before thermal annealing.

We then analyzed the thermodynamically favored PA system by using ThT as a fluorescence probe. The bundling of annealed PA was studied by adding ATP (100 µm) without any ATPase in the solution. The emission intensity immediately increases because of ATP‐regulated fibril formation and then plateaus (Figure S19, Supporting Information, green trace). While in the presence of ATPase enzyme (0.10 U mL^−1^) and without re‐annealing, the emission remains almost constant, contrary to the decay observed for the freshly prepared PA under the same conditions (Figure S19, Supporting Information, red trace). This observation is consistent with the experiments discussed above and the previous report by Prins and colleagues.^[^ ^95^ ^]^ The system can achieve reversibility only when the superstructures are re‐annealed in the presence of ATPase, as ATP may be entrapped in the large stable superstructure and not accessible to the enzyme.

We also investigated the chemical reactivity of lysine residues toward the deacetylation reaction of *p*‐NPA after thermal annealing of PA. In the presence of the thermodynamically favored PA nanofibers and without any ATP, after adding *p*‐NPA (500 µm) absorption traces show that *p*‐NP is produced in lower amounts when compared to freshly prepared PA under the same conditions (Figure S20, Supporting Information purple traces). Consistent with the microscopy evidence presented above, this indicates that the longer thermodynamically favored nanofibers implicate fewer lysine residues available for deacetylation of *p*‐NPA. Then, we studied the reactivity in the presence of PA superstructures, obtained by adding ATP (30 µm) to a solution containing *p*‐NPA (500 µm) and annealing PA (60 µm), without ATPase present. Even in this case, a rapid increase in absorbance (after *p*‐NPA addition) is followed by a downregulation of the deacetylation reaction, since the production of *p*‐NP almost plateaus (Figure S20, Supporting Information, green traces). While in the presence of ATPase enzyme (1.0 U mL^−1^) and without re‐annealing, the downregulation of *p*‐NPA deacetylation is followed by a smaller absorbance increase than the one observed for the freshly prepared PA under the same conditions (Figure S20, Supporting Information, red traces). This observation suggests that ATPase is hydrolyzing some ATP leading to a slight recovery of *p*‐NP production, however, since this system does not show reversibility without a re‐annealing process, the amount of *p*‐NP produced is noticeably lower than the one observed with the reversible system‐based on freshly prepared PA nanofibers.

## 3 Conclusion

In summary, we developed a system based on peptide amphiphile C_16_V_3_A_3_K_3_ that can transiently form hierarchical self‐assemblies of supramolecular nanofibers in solution. Polydisperse short nanofibers are observed when PA is freshly dissolved, while nanofiber bundles form after adding ATP. Microscopic and spectroscopic studies proved that the system is reversible when ATPase is present in solution to consume ATP, as the system reconverts to its original state of individualized nanofibers. Fluorescence kinetic experiments (using ThT) demonstrated the repeatability of the process by multiple and consecutive ATP additions. The transient performance of our PA‐based system was also employed to control over time the chemical reactivity (*p*‐NPA deacetylation reaction) and catalysis (methodol retro‐aldol reaction) of the lysine residues of PA. However, when thermodynamically favored PA nanofibers are used instead of the freshly prepared ones, a re‐annealing process in the presence of ATPase is necessary to reconvert the ATP‐templated superstructures into the initial stage. This suggests that different energetic states of PA nanostructures can influence the reversibility of the system, probably due to an increase in stability of the superstructure which leads to the ATP being buried and not accessible to the enzyme.

Future studies could focus on lysine reactivity and catalytic activity through molecular modeling both for freshly dissolved and thermally annealed PA systems. Furthermore, other (catalytic) reactions where lysines have been shown to work, like condensation reactions through imine bonds formation and successive transamination reactions, could also be applied to this system.^[^ ^96^ ^]^

Although we have focused on C_16_V_3_A_3_K_3_ peptide amphiphile, we envision that the same strategy may also be applied to other monomers. Tuning the peptide sequences could lead to different morphological changes and hierarchical self‐assemblies, but also to improved reactivity and catalysis. For example, histidine residues containing imidazoles are interesting candidates for tuning the PA sequence since they are widely exploited as nucleophiles to promote hydrolysis.^[^ ^88^, ^96^, ^97^, ^98^, ^99^, ^100^ ^]^ Similarly, the hydrophobic tail could be substituted with other hydrophobic groups. Likewise, tails carrying functional moieties, such as chromophores for example, could also increase the versatility of this straightforward method, leading to ATP‐templated superstructures that reversibly switch to the original state and that exhibit other properties to be controlled over time. Modification of the peptide sequence could also improve the reported low biocompatibility of the cationic C_16_V_3_A_3_K_3_ peptide amphiphile. For instance, incorporating either epitopes (such as the RGDS, Arg‐Gly‐Asp‐Ser) or stabilizing motifs into the monomer, are already proven routes toward improved biocompatibility of lysine‐containing peptides.^[^ ^101^, ^102^ ^]^ Therefore, the transient formation of peptide superstructures could be taken advantage of in the context of bioactivity of peptide‐based nanostructures, such as enzyme‐triggered release of hydrophobic small molecule drugs, or for their antimicrobial activity.^[^ ^102^, ^103^ ^]^

## Conflict of Interest

The authors declare no conflict of interest.

## Supporting information



Supporting Information

## Data Availability

The data that support the findings of this study are available from the corresponding author upon reasonable request.
