# Supplementary material for: ATP‐Regulated Formation of Transient Peptide Amphiphiles Superstructures
Source: Small. 2025 Feb 25;21(13):2410850. doi: 10.1002/smll.202410850 (PMC11962707; doi:10.1002/smll.202410850)
Supplement: Supplementary file 1 — Supporting Information [file SMLL-21-2410850-s001.pdf]

NANO · MICRO
small

Supporting Information

for *Small*, DOI 10.1002/smll.202410850

# ATP-Regulated Formation of Transient Peptide Amphiphiles Superstructures

*David Cappelletti, Federico Lancia, Andrea Basagni and Luka Đorđević**

Supporting Information

# ATP-regulated Formation of Transient Peptide Amphiphiles Superstructures

*David Cappelletti, Federico Lancia, Andrea Basagni, and Luka Đorđević**

**Table of Contents**

Materials ........ 1
Instruments and Methods ........ 2
Synthetic Procedures ........ 6
Supplementary Figures ........ 7
References ........ 25

## Materials

All commercially available reagents and chemicals were purchased and used as received from Merck-Sigma Aldrich, TCI chemicals, Fisher Scientific, and VWR. Solvents were purchased and were used without further purifications from Sigma Aldrich and ThermoFisher. Deuterated solvents were bought from Sigma Aldrich. The enzyme potato apyrase (A6237-100UN) was obtained from Sigma Aldrich and used without further purification – it was dissolved in 1.0 mL of mQ-water, divided into 25 working aliquots of 40 μL each with a concentration of 100 U $mL^{-1}$, and preserved at −20 °C. The fluorescence probe ThT was obtained from TCI chemicals and used as received. Even in this case, a ThT 1.0 mM solution in mQ-water was prepared, divided into working aliquots, and stored at −20 °C. HEPES (4-(2-hydroxyethyl)-1-piperazineethanesulfonic acid) was obtained from Fisher Scientific (Fisher BioReagents BP310-1). The plate used for kinetic experiments was a Greiner CELLSTAR® 96-well plate (flat-bottom black polystyrene wells with micro-clear bottom).

# Instruments and Methods

**NMR spectroscopy.** NMR spectra were obtained on a Varian 400 MHz NMR spectrometer at room temperature (298 K). Chemical shifts are reported in ppm using the solvent residual signal as internal reference (Chloroform-*d*: δH = 7.26 ppm). The resonance multiplicity is described as s (singlet), d (doublet), t (triplet), …, m (multiplet), dd (doublet of doublets), bs (broad singlet).

**Column chromatography.** Column chromatography was performed using a Buchi Pure C-810 Flash automated system with pre-packed FlashPure Buchi irregular 50 µm silica gel.

**Liquid chromatography–mass spectrometry (HPLC-MS).** HPLC (Agilent Technologies 1100 series) equipped with a mass spectrometer detector (MSD Trap SL, model G2245D) with ESI source was used. The ionization was performed in positive ion mode under the following conditions: nebulizer pressure of 65 psi, dry gas flow rate of 12 L min$^{-1}$, dry gas temperature of 350 °C, capillary voltage of 3.5 kV, capillary exit voltage of 177.3 V, and skimmer voltage of 40 V. Chromatographic separation was carried out on a Poroshell 120 EC-C18 column (100 mm x 4.6 mm i.d., particle size 4 µm). The eluents were mQ-water (A) and acetonitrile (B) both containing 0.1% TFA. The gradient elution for B was programmed as follows: from 10% to 90% over 30 minutes, followed by 5 minutes of isocratic elution. The flow rate was set to 0.8 mL min$^{-1}$. Two sample solutions were prepared: (1) 100 µM PA in mQ-water for the evaluation of the peptide purity, and (2) 100 µM PA and 500 µM *p*-nitrophenylacetate (*p*-NPA) in HEPES buffer to assess the mechanism of the deacetylation reaction. Sample (1) was diluted and injected, while sample (2) was diluted after 30 minutes to let the reaction occur and then injected. The volume of injections was 10 µL.

**UV-Vis spectroscopy.** Absorption spectra were recorded at 25 °C with an Agilent Cary 60 UV-Vis spectrophotometer, using reduced-volume quartz cells with a path length of 1.0 cm. Three sample solutions were prepared in Eppendorf tube® and transferred into the cuvette (filled with about 270 µL): PA (100 µM) in HEPES buffer, PA (100 µM)-ATP (60 µM) in HEPES buffer (ATP added from a stock solution in mQ-water), and PA (100 µM)-enzyme (0.50 U mL$^{-1}$)-ATP (60 µM) (ATP added from a stock solution in mQ-water to PA-enzyme solution). The sample solutions containing enzyme were allowed to shake at room temperature for 1 h before being transferred into the cuvette for analysis.

**Fluorescence spectroscopy.** Fluorescence emission spectra were recorded at 25 °C on an Agilent Cary Eclipse fluorescence spectrofluorometer, using reduced-volume quartz cells with a path length of 1.0 cm. Three sample solutions containing the fluorescence probe ThT were

prepared using the same procedure reported above for UV-Vis spectroscopy. As excitation wavelength was chosen 440 nm and slit width (ex./em.) = 5/5 nm. Fluorescence experiments with PA (100 μM), ThT (10 μM), and different concentrations of ATP/ADP/AMP/AMP+2$P_i$ (0-120 μM) were recorded using a Tecan Spark® plate reader and a 96-multiwell plate at 30 °C. The sample solutions were prepared in Eppendorf tube® and transferred to the plate with micropipettes (final volume of 200 μL per well). Then the plate was placed inside the instrument for analysis. Fluorescence top reading mode was selected, as excitation wavelength was chosen 440 nm (bandwidth of 20 nm), and the emission wavelength was recorded at 483 nm (bandwidth of 20 nm).

**Time-dependent absorption and fluorescence readings.** *Kinetic UV-Vis studies* were performed with a Tecan Spark® plate reader and using a 96-multiwell plate at 30 °C. The sample solutions were prepared in Eppendorf tube®, transferred to the plate with micropipettes, and then the plate was placed inside the instrument for 10 minutes before starting the kinetics (monitored absorbance at 400 nm). Additions of *p*-nitrophenylacetate (*p*-NPA) in DMF and ATP in mQ-water were performed through separated automatic injections directly in the Tecan Spark® plate reader. *p*-NPA in DMF (10 mM, 10 μL) and ATP in mQ-water (1.0 mM, 0-18 μL) were added to each well at 0 and 100 seconds, respectively. If necessary, the third addition (as in case of enzyme in Figure 4c) was performed by taking the plate out of the instrument for 30 seconds and adding the desired amount with a micropipette. In general, a final volume of 200 μL per well was reached after the additions. During the entire kinetics, before each absorption reading cycle, the plate was allowed to shake for 15 seconds. Where ATP is added and thus PA superstructures formed, to remove the scattering given by the aggregates, the reported absorbances (Figure 4b-d, Figures S14, and S20) were corrected by subtracting the initial absorbances of PA-ATP control solutions (without *p*-NPA). While, where ATP is not added, the reported absorbances (Figure 4b-d, Figures S12, S14, and S20) were corrected by subtracting the absorbances of PA control solutions (without *p*-NPA and ATP) over time. *Kinetic fluorescence studies* were performed with the same instrument in the same experimental and sample preparation conditions. In particular, in this case, for the ThT fluorescence studies, an ATP stock solution in mQ-water (1.0 mM, 0-20 μL) and mQ-water were simultaneously added to the wells to achieve the desired ATP concentration per well. Fluorescence top reading mode was selected, as excitation wavelength was chosen 440 nm (bandwidth of 20 nm), and the emission wavelength was recorded at 483 nm (bandwidth of 20 nm). Whereas, for the organocatalytic studies, a stock solution of methodol in DMF (10 mM, 10 μL) and one of ATP in mQ-water (1.0 mM, 0-16 μL) were added to the wells (at 0 and 100 s, respectively) to obtain

the desired amount of ATP and 500 μM of methodol for each well. Fluorescence top reading mode was selected, as excitation wavelength was chosen 330 nm (bandwidth of 20 nm), and the emission wavelength was recorded at 460 nm (bandwidth of 20 nm). Even in kinetic fluorescence studies, during the entire kinetics, before each absorption reading cycle, the plate was allowed to shake for 15 seconds.

**Circular dichroism (CD) spectroscopy.** CD spectra were recorded at 25 °C on a Jasco J-1500 instrument, using quartz cells with a path length of 0.50 mm. Spectra were recorded from 190 to 300 nm (step size 1 nm). Two sample solutions were prepared in Eppendorf tube®: 100 μM PA and 100 μM annealed PA in HEPES buffer. The cuvettes were filled with about 40 μL of sample solution.

**Dynamic light scattering (DLS) and Zeta-potential (*ζ*-potential).** *DLS* was performed on a Malvern Zetasizer Nano-ZS instrument at 25 °C, using disposable microcuvettes. Three sample solutions were prepared in Eppendorf tube® and transferred into the microcuvette (filled with about 60 μL): PA (100 μM) in HEPES buffer, PA (100 μM)-ATP (60 μM) in HEPES buffer (ATP added from a stock solution in mQ-water), and PA (100 μM)-enzyme (10 U $mL^{-1}$)-ATP (60 μM) (ATP added from a stock solution in mQ-water to PA-enzyme solution). To favor the complete reversibility of PA superstructures, a higher enzyme concentration (10 U $mL^{-1}$) was used when compared to fluorescence and UV-Vis experiments. Moreover, the sample solution containing enzyme was allowed to shake at room temperature for 1 h, then sonicated for 2 minutes, before being transferred into the microcuvette for analysis. Measurements were recorded selecting water as dispersant (viscosity $\eta$ = 0.8872, refractive index $n$ = 1.33), protein as material (RI = 1.45), 173° as measurement angle, and 2 minutes as equilibration time. One measurement consists of 11 runs (10 s each), and each sample was measured 3 times. *Zeta-potential* was performed with the same instrument in the same experimental conditions, using a Folded Capillary Zeta Cell. Samples were prepared in Eppendorf tube® and transferred into the cell (filled with about 800 μL) after washing it with the buffer solution each time. Measurements were recorded selecting water as dispersant (viscosity $\eta$ = 0.8872, refractive index $n$ = 1.33), protein as material (refractive index $n$ = 1.45), Smoluchowski as model, 2 minutes as equilibration time, and Auto mode as analysis model. One measurement consists of 40 runs, and each sample was measured 3 times.

**Transmission electron microscopy (TEM).** TEM was performed with a Jeol 300PX instrument. Three sample solutions were prepared using the same preparation procedure reported above for DLS. Then, a glow-discharged carbon-coated grid was first placed on a drop of sample solution for 1 min, then on a drop of 2% uranyl acetate solution for 1 min, on a drop

of mQ-water for 30 s to wash away dissolved salts and then blotted with filter paper. The stained sample was allowed to dry before imaging. Images were analyzed using Image J software.[1]

**Atomic force microscopy (AFM).** AFM images were recorded with an Agilent 5500 Scanning Probe Microscope equipped with an Agilent N9521A scanner. The measurements were performed in tapping mode using cantilever C (force constant: 7 N/m) of MikroMash XSC11 Series. Sample solutions containing freshly dissolved PA or annealed PA were prepared using the same preparation procedure reported above for DLS. However, in the case of samples with annealed PA containing ATP and enzyme, after shaking for 1 h, one was sonicated (2 mins) and the other one was not. Then, a small volume (50 μL) of sample solution was placed on mica after peeling it off with tape. The excess solution was removed with compressed air after 1 min, and then 50 μL of mQ-water was placed on the mica to wash away dissolved salts. After 30 s, water was removed with compressed air and the mica was allowed to dry before imaging. Images were analyzed using Gwyddion software.[2]

**Scanning electron microscopy (SEM).** SEM was performed with a Zeiss Sigma HD microscope, equipped with a Schottky FEG source at 5 kV power and an InLens detector. Sample preparation involved PA-ATP solution (1.0 mM of ATP and peptide) drop-casting on a silicium plate, letting it dry, and then analysis.

**Scanning electron microscopy (SEM) with energy dispersive X-ray spectroscopy (EDX).** The instrument used is an Environmental Scanning Electron Microscopy (ESEM), FEI Quanta 200 model with tungsten filament as source at 20 kV. EDX analysis and maps were acquired with an Oxford Xplore 30 model as EDX detector and using Oxford Aztec Live as software. All the analyses were performed at CEASC of University of Padua. Two samples were prepared: annealed PA (1.0 mM)-ATP (1.0 mM) in HEPES buffer and annealed PA (1.0 mM)-enzyme (10 U $mL^{-1}$)-ATP (1.0 mM) (enzyme added from a stock solution in mQ-water to annealed PA-ATP solution). After shaking the samples for 1h, each solution was drop-casted on a germanium plate and let dry (repeated 4 times to acquire good EDX analysis). Before analysis, a drop of mQ-water was used to wash away dissolved salts and blotted with filter paper.

**Thermal Annealing.** Thermal annealing was performed with a Fishebrand™ Isotemp™ Water Bath, set to incubate a PA stock solution (0.12 mM for microscopy, spectroscopic, and reactivity studies or 1.2 mM for SEM-EDX analysis) in buffer at 80 °C for 30 minutes, then turning off the heating and letting the bath naturally cool down overnight (referred to as slow cooling in the main text). The annealed PA solution was used the following day.

# Synthetic Procedures

**Synthesis of $C_{16}V_3A_3K_3$ peptide amphiphile.** The peptide was synthesized according to previously reported protocols.[3] Its purity was determined by analytical high-performance liquid chromatography (HPLC) and electrospray ionization mass spectrometry in positive mode (Figure S1).

**Synthesis of 4-hydroxy-4-(6-methoxy-2-naphthyl)-2-butanone (methodol).** Methodol was prepared following a published procedure via condensation of acetone with 6-methoxy-2-naphthaldehyde.[4] In a pre-heated round bottom flask, L-proline (185 mg, 1.61 mmol) was stirred in dry DMSO/acetone (4:1 *v*/*v*, 70 mL) for 30 mins. 6-Methoxy-2-naphthaldehyde (1.00 g, 5.37 mmol) was added and the mixture was stirred for 16 h. Then, the mixture was treated with a saturated aqueous ammonium chloride solution and extracted with ethyl acetate. The collected organic phase was dried over $Na_2SO_4$, filtered, and concentrated to dryness. The crude product was purified by column chromatography (hexane-ethyl acetate with a gradient of 100% hexane → 40% hexane, 60% ethyl acetate). All pure fractions were collected and dried under reduced pressure to give a yellow solid as product (454 mg, 35% yield).

$^1$H NMR (400 MHz, Chloroform-*d*): δ = 7.75 – 7.71 (m, 3H), 7.44 – 7.42 (dd, $J$ = 8.6 Hz, $J$ = 1.4 Hz, 1H), 7.17 – 7.14 (m, 2H), 5.31 – 5.28 (m, 1H), 3.92 (s, 3H), 3.31 (d, $J$ = 3.01 Hz, 1H), 3.01 – 2.87 (m, 2H), 2.21 (s, 3H).

**Synthesis of *p*-nitrophenylacetate (*p*-NPA).** *p*-NPA was prepared following a published procedure via acetylation with acetic anhydride.[5] A solution of 4-nitrophenol (3.0 g, 21.6 mmol) in pyridine (4 mL, 49.4 mmol) was treated with acetic anhydride (4 mL, 42.3 mmol). The mixture was stirred at room temperature for 16 h. Then, 5% hydrochloric acid (20 mL) was added, and the mixture was stirred for 2 h. The aqueous phase was extracted with methylene chloride, the combined organic phase was washed with 5% HCl and then water. The collected organic phase was dried over $Na_2SO_4$, filtered, and concentrated to dryness. The crude product was purified by column chromatography (petroleum ether-ethyl acetate with a gradient of 100% petroleum ether → 70% petroleum ether, 30% ethyl acetate). All pure fractions were collected and dried under reduced pressure to give a white solid as product (3.12 g, 80% yield).

$^1$H NMR (400 MHz, Chloroform-*d*): δ = 8.27 (d, $J$ = 9.1 Hz, 2H), 7.29 (d, $J$ = 9.1 Hz, 2H), 2.35 (s, 3H).

# Supplementary Figures

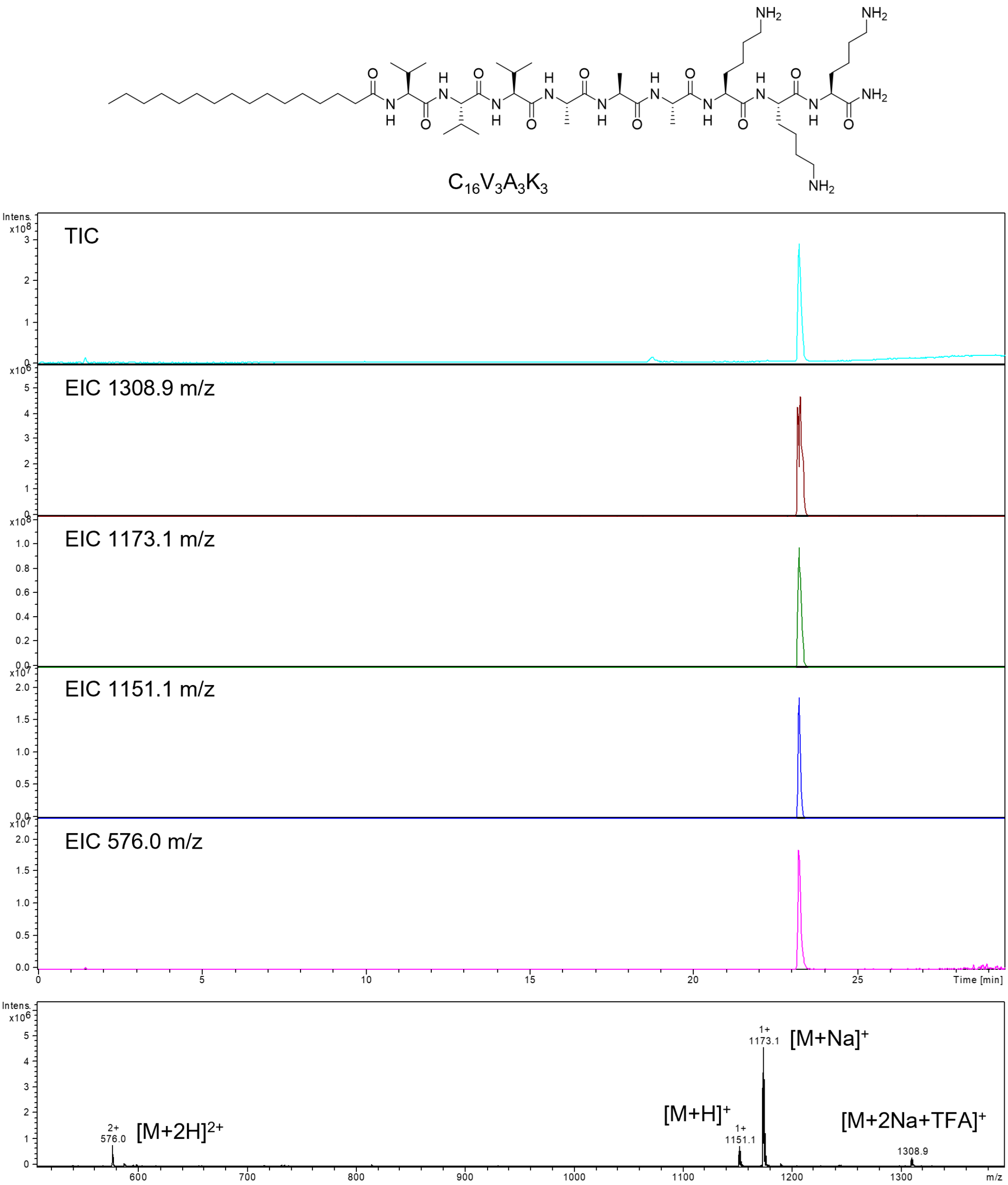


**Figure S1**. Chemical structure of $C_{16}V_3A_3K_3$ PA used in this work and assessment of its purity through HPLC-MS chromatograms.

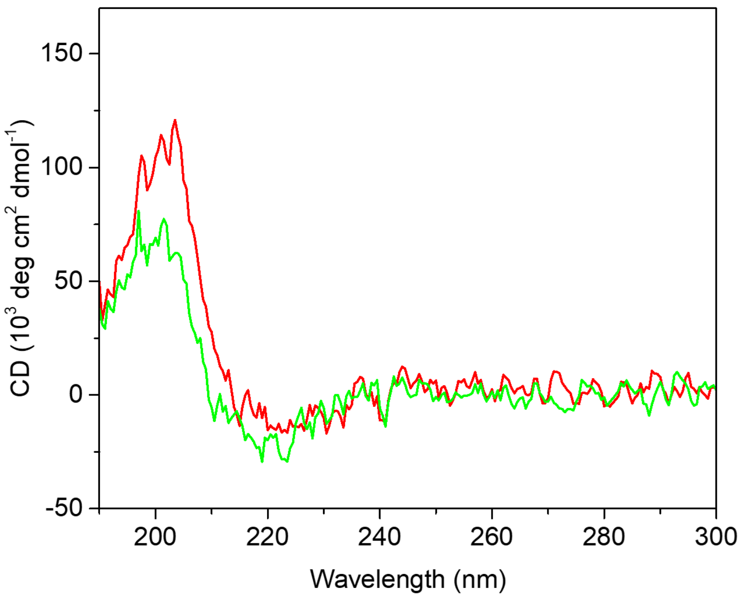


**Figure S2.** Comparison between CD spectra of freshly dissolved (green) and annealed PA (red) at the same concentration (100 µM). A greater β-sheet contribution is observed after the thermal annealing process of an aqueous buffer solution of PA. Experimental conditions: HEPES (10 mM, 7.2 pH), $CaCl_2$ (1.0 mM), T = 25 °C.

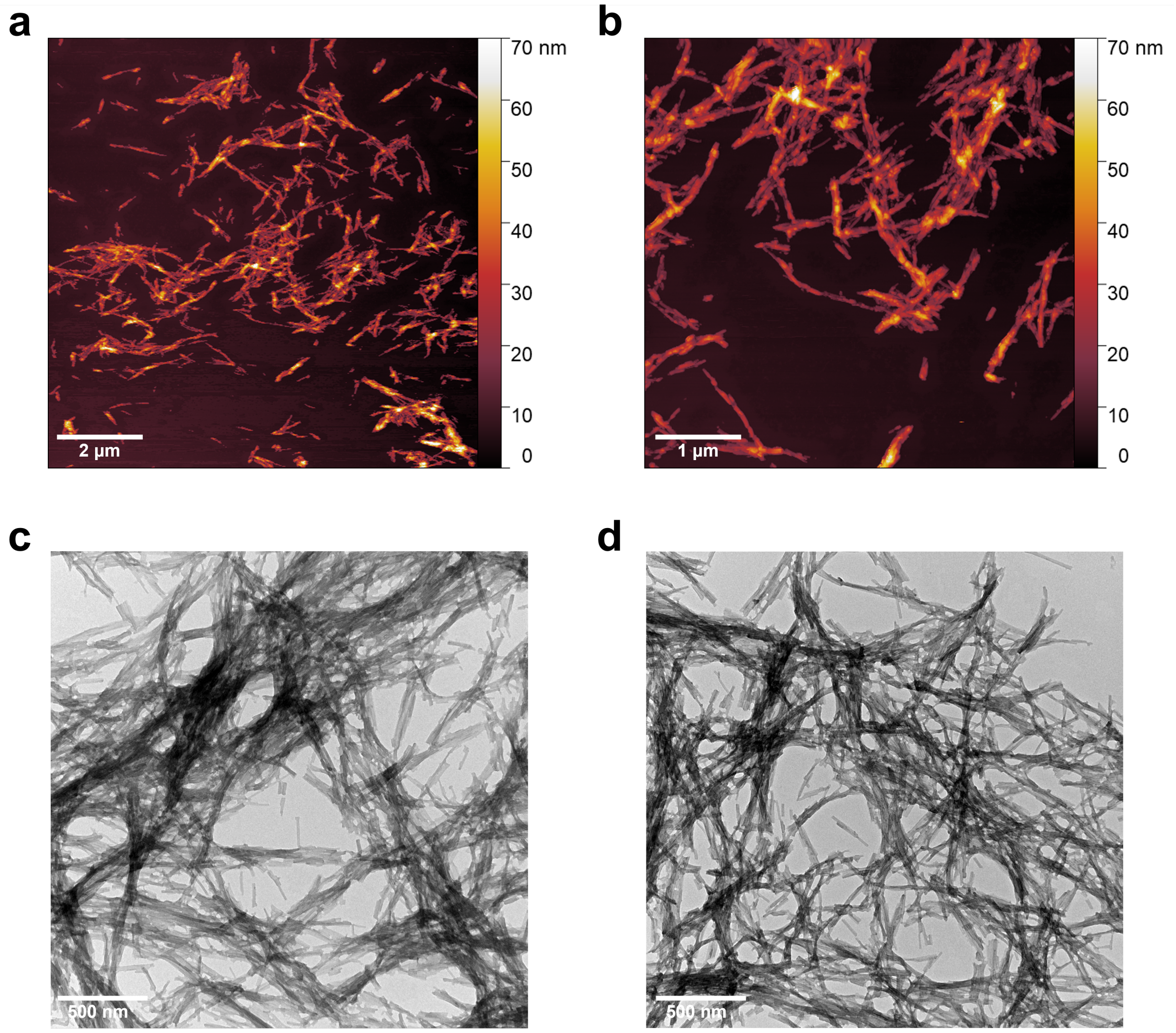


**Figure S3.** AFM (a,b) and TEM (c,d) micrographs of PA-ATP superstructures. Experimental conditions: $C_{16}V_3A_3K_3$ (100 µM), ATP (60 µM), HEPES (10 mM, 7.2 pH), $CaCl_2$ (1.0 mM).

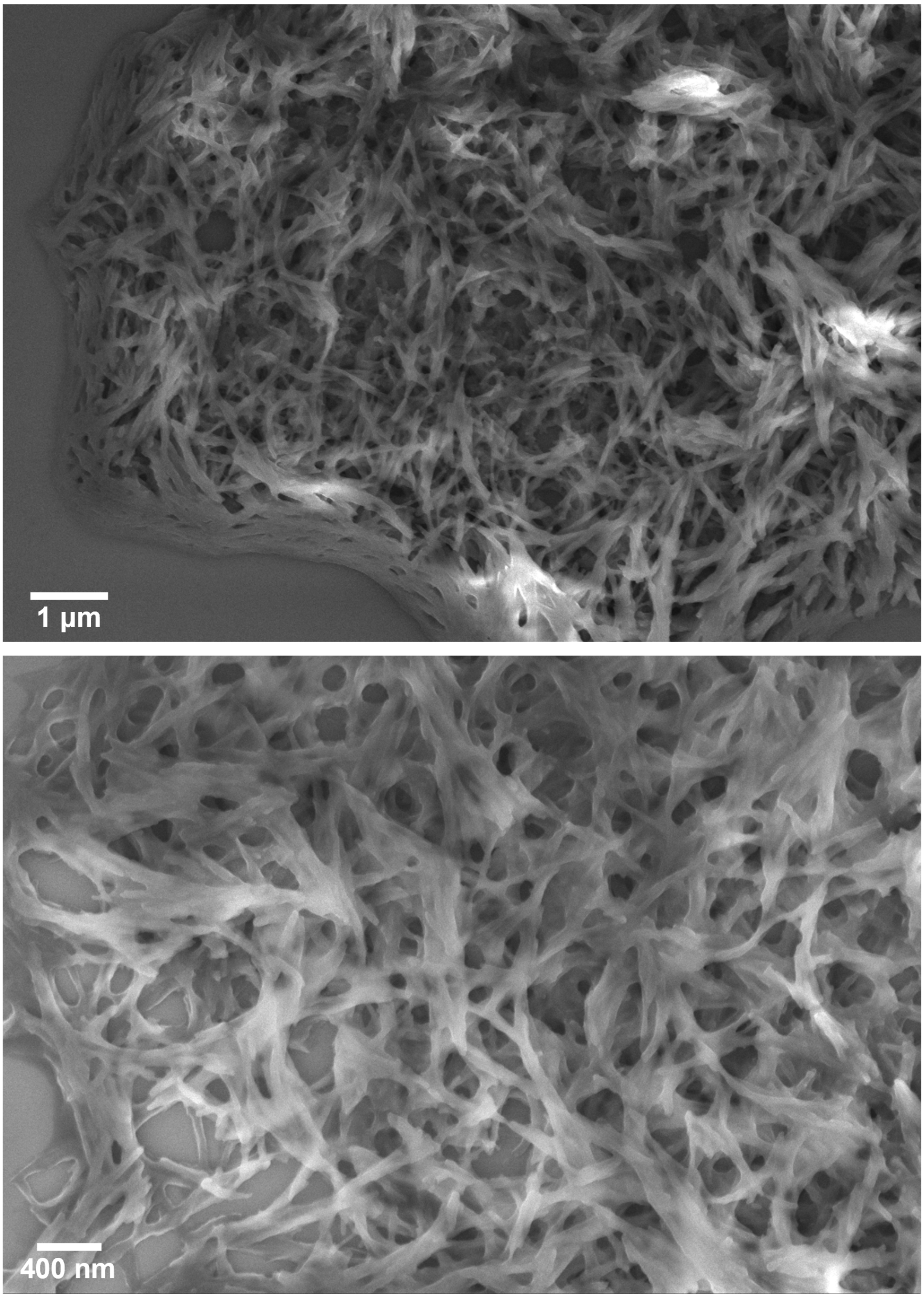


**Figure S4.** SEM images of PA-ATP superstructures (1.0 mM of ATP and peptide) showing a network of bundled fibers. Experimental conditions: HEPES (10 mM, 7.2 pH), $CaCl_2$ (1.0 mM).

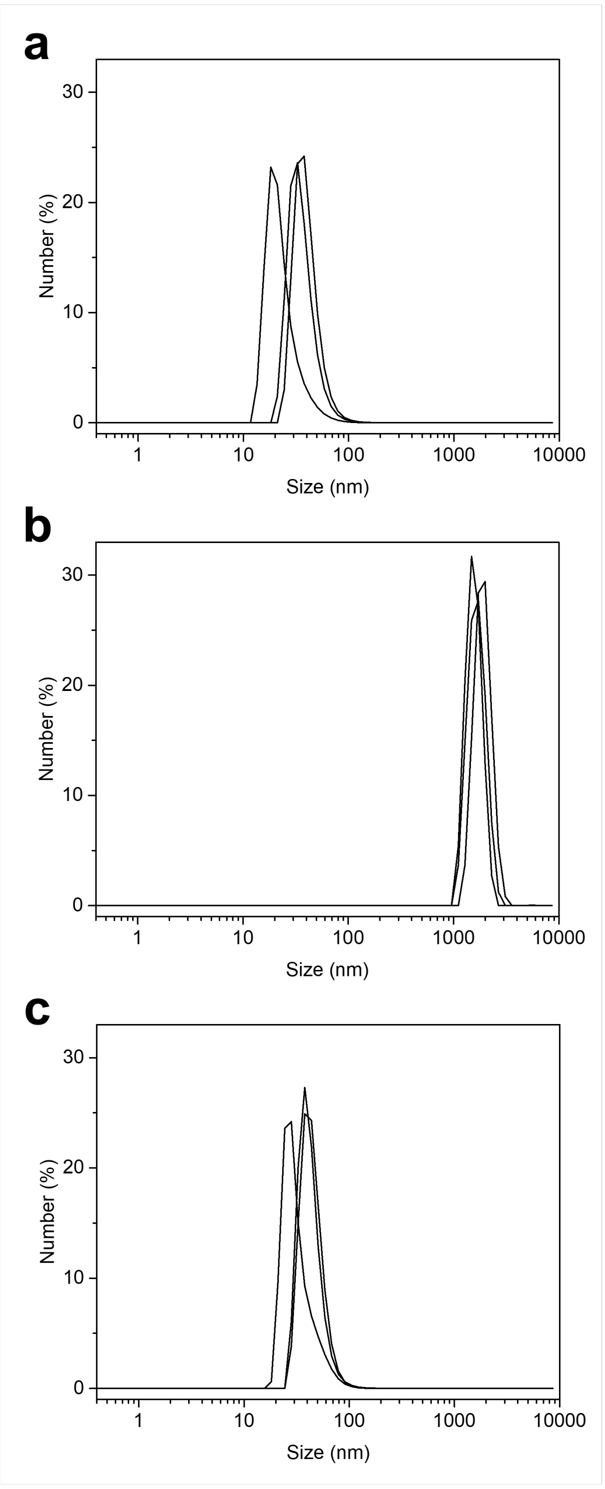


**Figure S5.** DLS measurements (3 measurements per sample) of solutions containing a) PA (100 µM) in the absence and b) presence of ATP (60 µM). We note a large increase in the hydrodynamic diameter after adding ATP and c) the reconversion to the original size in the presence of enzyme (10 U mL$^{-1}$). Experimental conditions: $C_{16}V_3A_3K_3$ (100 µM), HEPES (10 mM, 7.2 pH), $CaCl_2$ (1.0 mM), T = 25 °C.

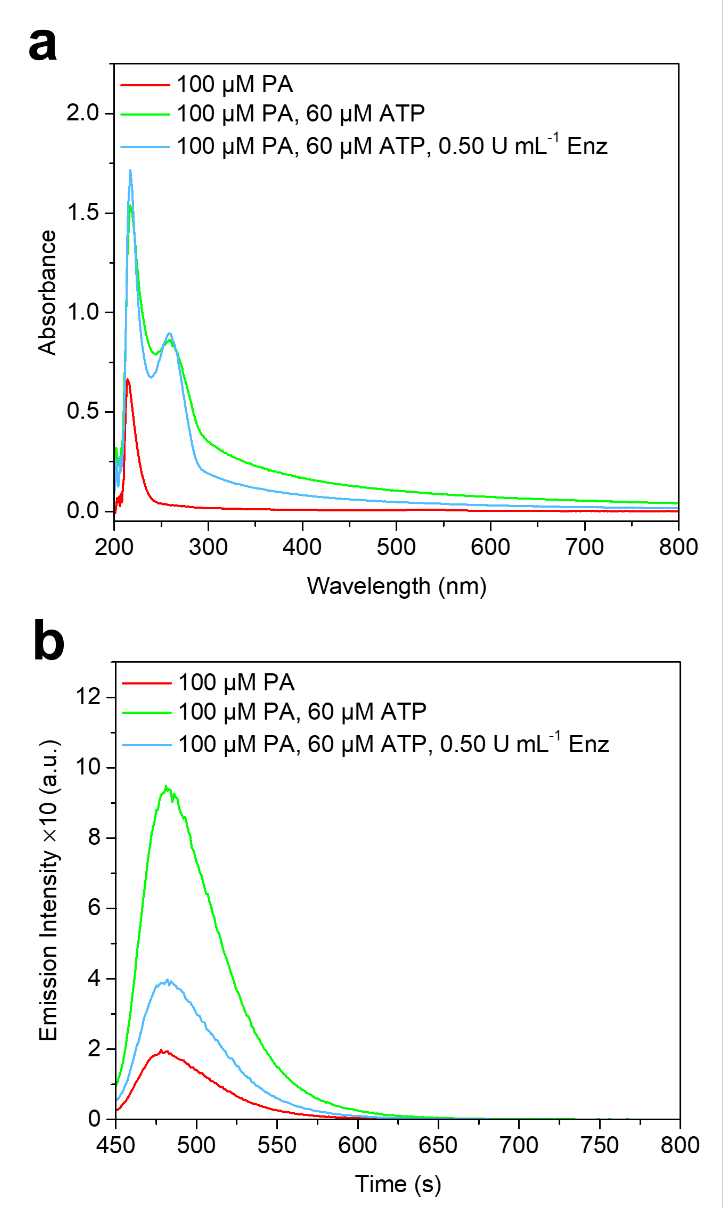


**Figure S6.** Three samples were analyzed by a) UV-Vis and b) fluorescence spectroscopy. The sample with only PA shows no scattering in the UV-Vis spectrum and emission of ThT in the fluorescence spectrum (red lines). Upon the addition of ATP (60 µM), the scattering and the emission increase due to the formation of superstructures (green lines). However, if potato apyrase (0.50 U mL$^{-1}$) is in solution, the absorbance and emission intensity decrease due to the disassembly of aggregates (blue lines). It is possible to notice that the three samples exhibited slightly different emission maxima (about a few nanometers) around 483 nm, possibly due to equilibration of ThT binding with fibrils.[6,7] Experimental conditions: $C_{16}V_3A_3K_3$ (100 µM), HEPES (10 mM, 7.2 pH), $CaCl_2$ (1.0 mM), T = 25 °C, ThT 10 µM ($\lambda_{ex.}$ = 440 nm) and slit width (ex./em.) = 5/5 nm for fluorescence experiments.

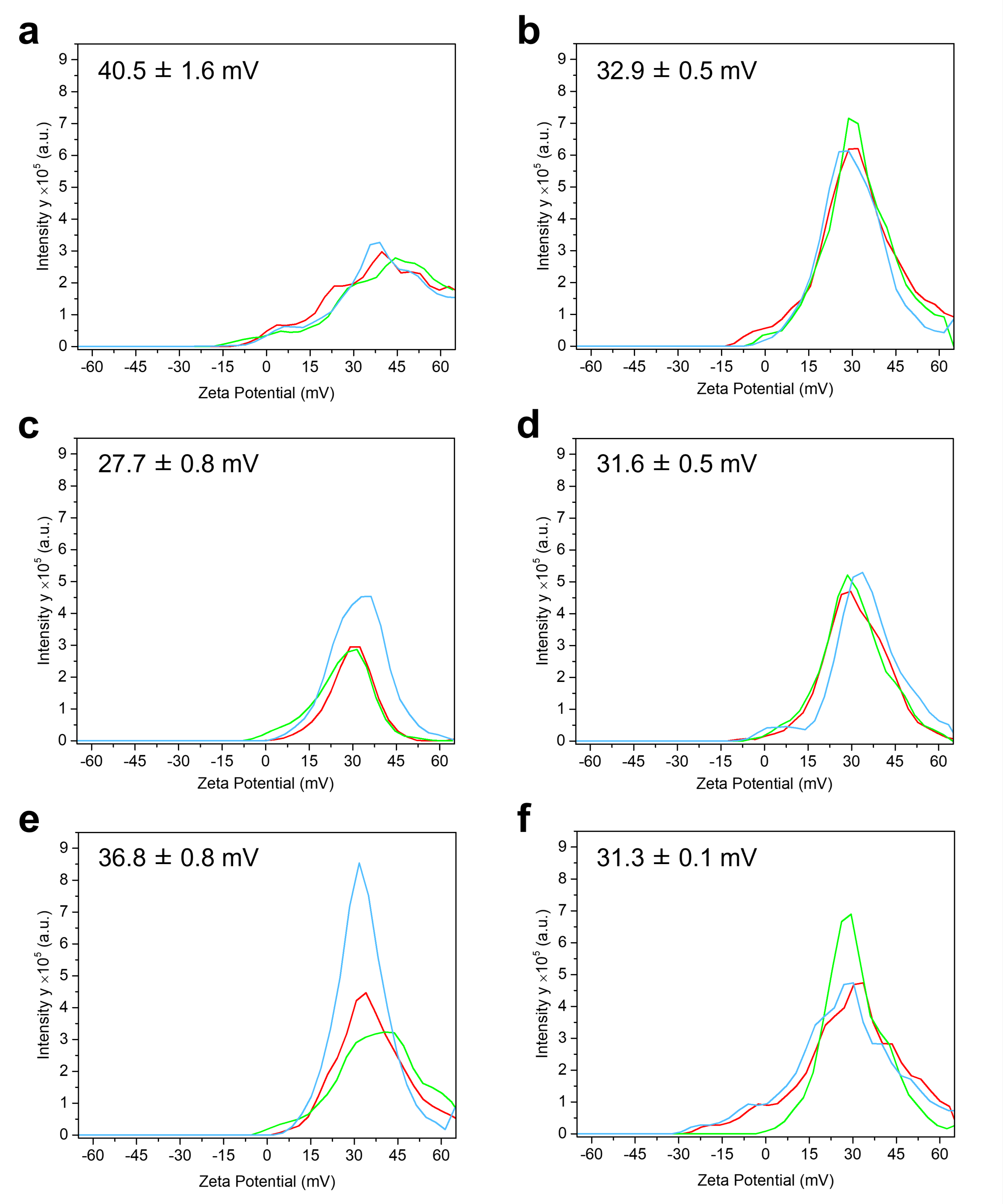


**Figure S7.** Zeta-potential measurements (3 measurements per sample) of solutions containing a) PA (100 µM) in the absence and b) presence of ATP (40 µM), c) ATP (60 µM), d) ADP (60 µM), e) AMP (60 µM), and f) AMP (120 µM). At the same concentration of adenosine phosphates (c,d,e), we notice that the surface charge decreased from PA (a) → PA+AMP (e)→ PA+ADP (d) → PA+ATP (c). Whereas, the surface charge remains constant at the same amount of negatively charged phosphate groups (b,d,f). Experimental conditions: $C_{16}V_3A_3K_3$ (100 µM), HEPES (10 mM, 7.2 pH), $CaCl_2$ (1.0 mM), T = 25 °C.

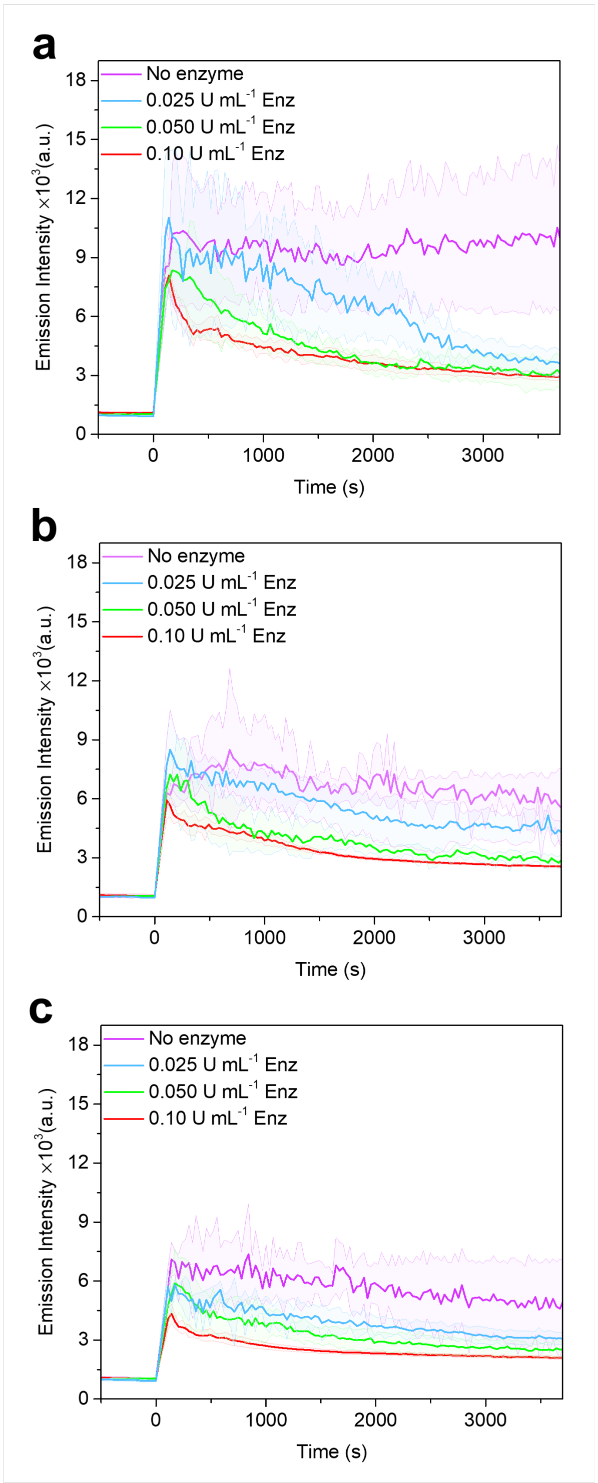


**Figure S8.** Kinetic fluorescence experiments with ThT (10 µM) as fluorescence probe where a) 100 µM, b) 80 µM, and c) 60 µM ATP were added at 0 s to solutions with 100 µM PA and diverse concentrations of potato apyrase. After ATP addition, the emission intensity of ThT increases and then gradually decayes when the enzyme is present in solution. In particular, the higher the amount of enzyme, the faster the decay. Experimental conditions: $C_{16}V_3A_3K_3$ (100 µM), HEPES (10 mM, 7.2 pH), $CaCl_2$ (1.0 mM), T = 30 °C, ThT 10 µM ($\lambda_{ex.}$ = 440 nm / $\lambda_{em.}$ = 483 nm). All the error bars denote standard deviation based on quadruplicate measurements.

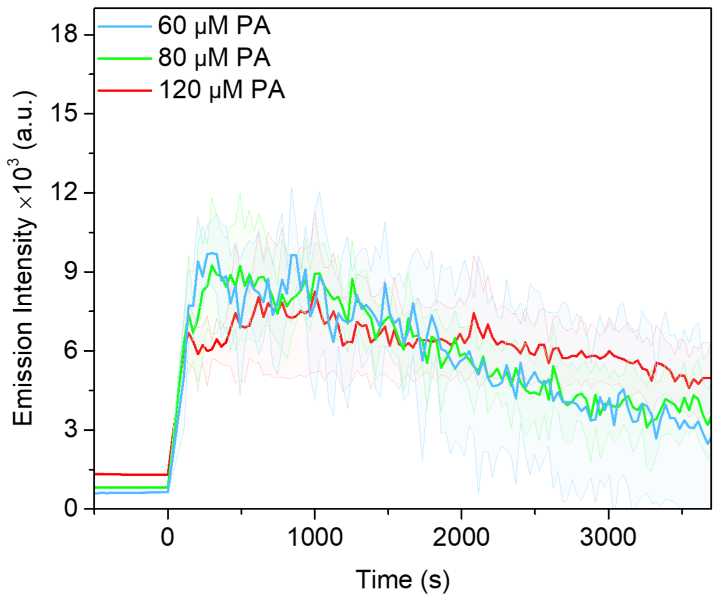


**Figure S9.** Kinetic fluorescence experiments to study the effect of diverse PA concentrations after the addition of ATP (80 µM) in the presence of potato apyrase (0.050 U mL$^{-1}$). Experimental conditions: HEPES (10 mM, 7.2 pH), $CaCl_2$ (1.0 mM), T = 30 °C, ThT 10 µM ($\lambda_{ex.}$ = 440 nm / $\lambda_{em.}$ = 483 nm). All the error bars denote standard deviation based on quadruplicate measurements.

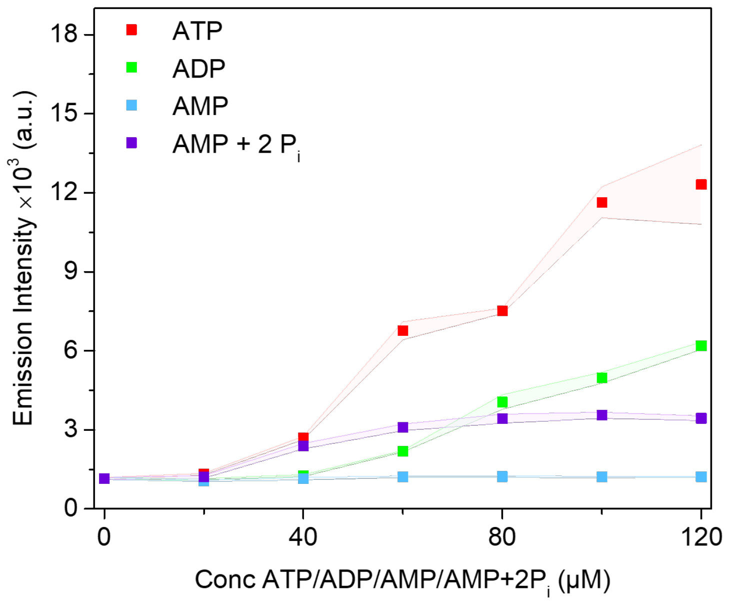


**Figure S10.** Fluorescence experiment to investigate the effect of ATP hydrolysis waste products on PA bundling. Different amounts AMP + 2 $P_i$ were added to aqueous buffer solutions containing only PA and ThT (purple trace). Red, green, and blue traces from Figure 4a were reported also in this figure for comparison. Same experimental conditions adopted for experiments in Figure 3a: $C_{16}V_3A_3K_3$ (100 µM), HEPES (10 mM, 7.2 pH), $CaCl_2$ (1.0 mM), 30 °C, ThT 10 µM ($\lambda_{ex.}$ = 440 nm / $\lambda_{em.}$ = 483 nm). All the error bars denote standard deviation based on quadruplicate measurements

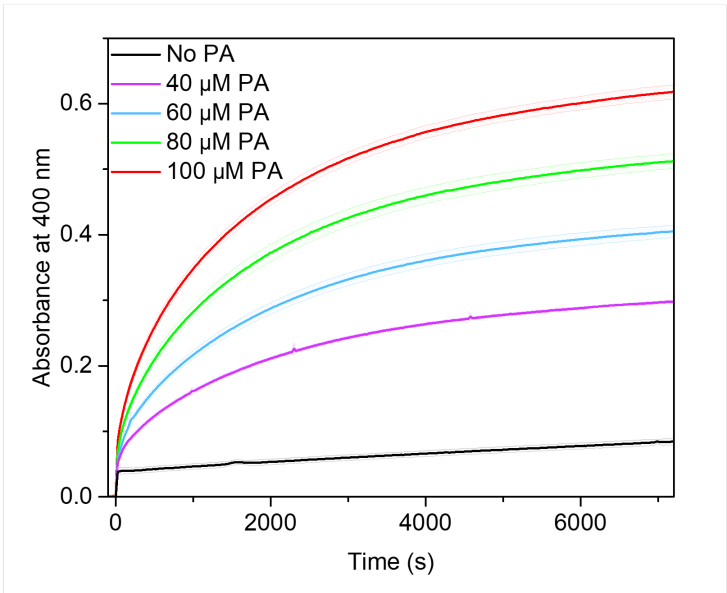


**Figure S11.** Kinetic experiments to study the reactivity of PA towards the model deacetylation reaction. *p*-NPA (500 μM) was added at 0 s to solutions with different amounts of PA and the reaction was followed by UV-Vis. Experimental conditions: HEPES (10 mM, 7.2 pH), $CaCl_2$ (1.0 mM), 5% DMF to dissolve *p*-NPA, T = 30 °C, $\lambda_{abs.}$ = 400 nm. All the error bars denote standard deviation based on quadruplicate measurements.

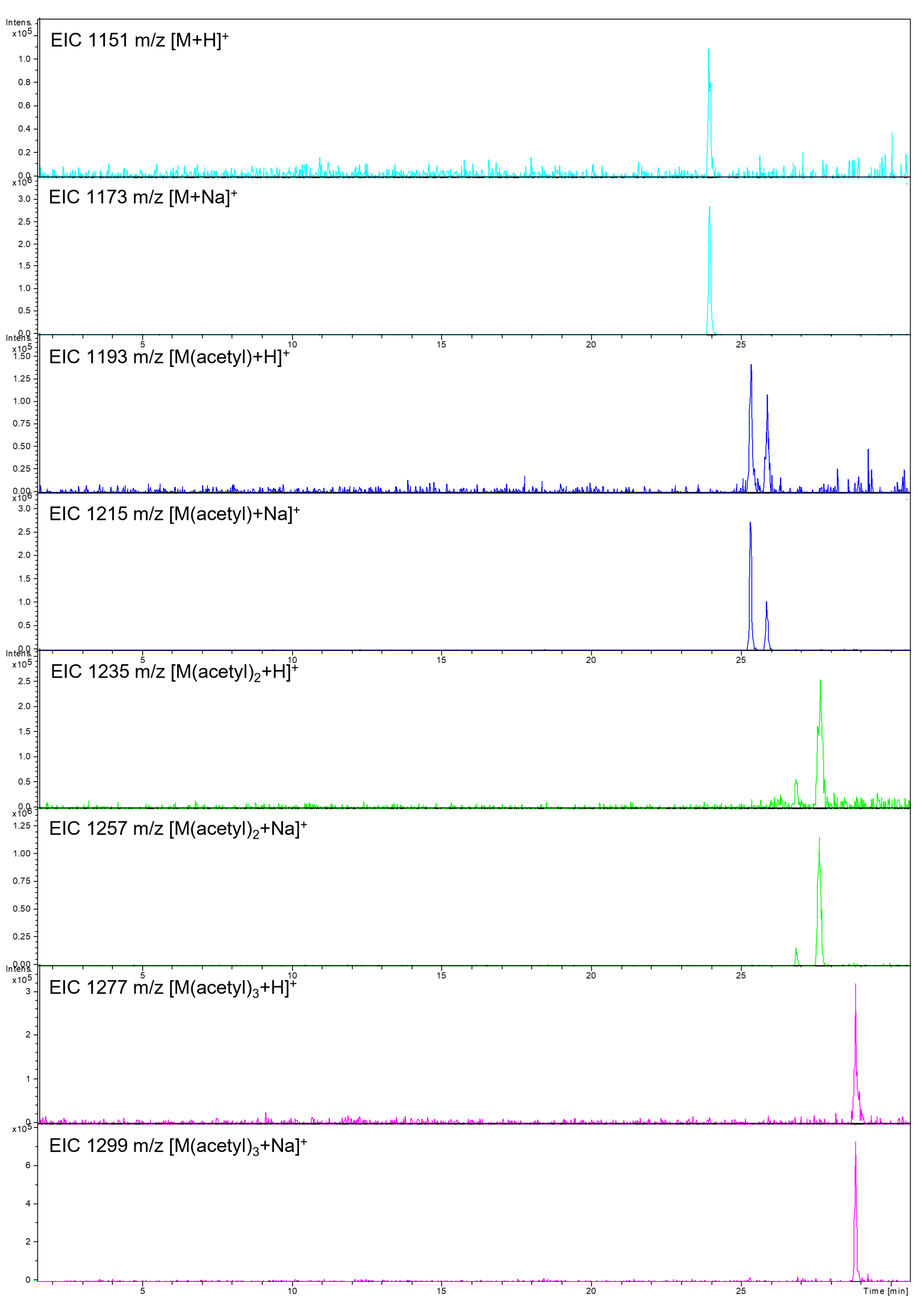


**Figure S12.** HPLC-MS chromatograms of PA cations after deacetylation reaction of 500 µM *p*-NPA in presence of 100 µM PA. Experimental conditions: HEPES (10 mM, 7.2 pH), $CaCl_2$ (1.0 mM), 5% ACN to dissolve *p*-NPA. Four species are found from analysis: (1) not acetylated PA (light blue chromatograms, ions 1151 and 1173 m/z), also detected in the HPLC-MS analysis reported in Figure S1, (2) mono-acetylated PA (blue chromatograms, ions 1193 and 1215 m/z), (3) bi-acetylated PA (green chromatograms, ions 1235 and 1257 m/z), and (4) tri-acetylated PA (pink chromatograms, ions 1277 and 1299 m/z). In particular, two isomers of (2) and (3) with different retention times are found depending on which lysines of PA are acetylated

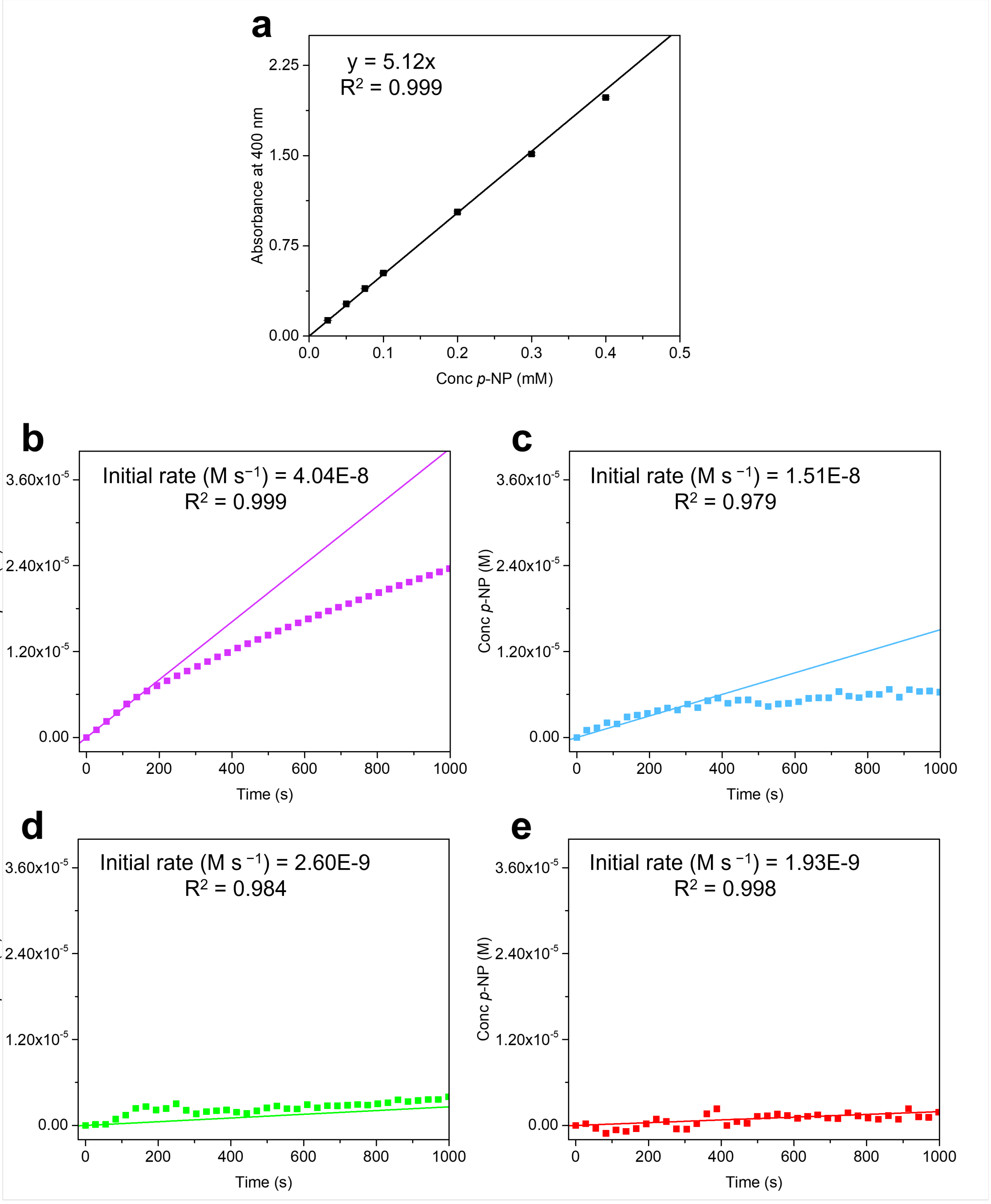


**Figure S13.** a) Calibration curve using *p*-NP (reaction product) standard solutions. Experimental conditions: HEPES (10 mM, 7.2 pH), $CaCl_2$ (1.0 mM), 5% DMF to dissolve *p*-NP, T = 25 °C, $\lambda_{abs.}$ = 400 nm. All the error bars denote standard deviation based on duplicate measurements. b,c,d,e) Comparison of initial rates (M $s^{-1}$) of *p*-NPA deacetylation reaction after b) no addition of ATP, c) addition of 30 µM, d) 60 µM, and e) 90 µM of ATP. The absorbances from 100 s to 1100 s of experiments in Figure 4a were elaborated so that the first absorbance value at 400 nm was set to zero, and then they were divided by the slope of the calibration curve (multiplied by 1000 for the conversion from mM to M). Therefore, the data from 0 to 100 s and from 1100 to 2500 s of Figure 4a are not considered here. The linear fitting was performed till 170 s (b), 400 s (c) or in the whole interval (d,e).

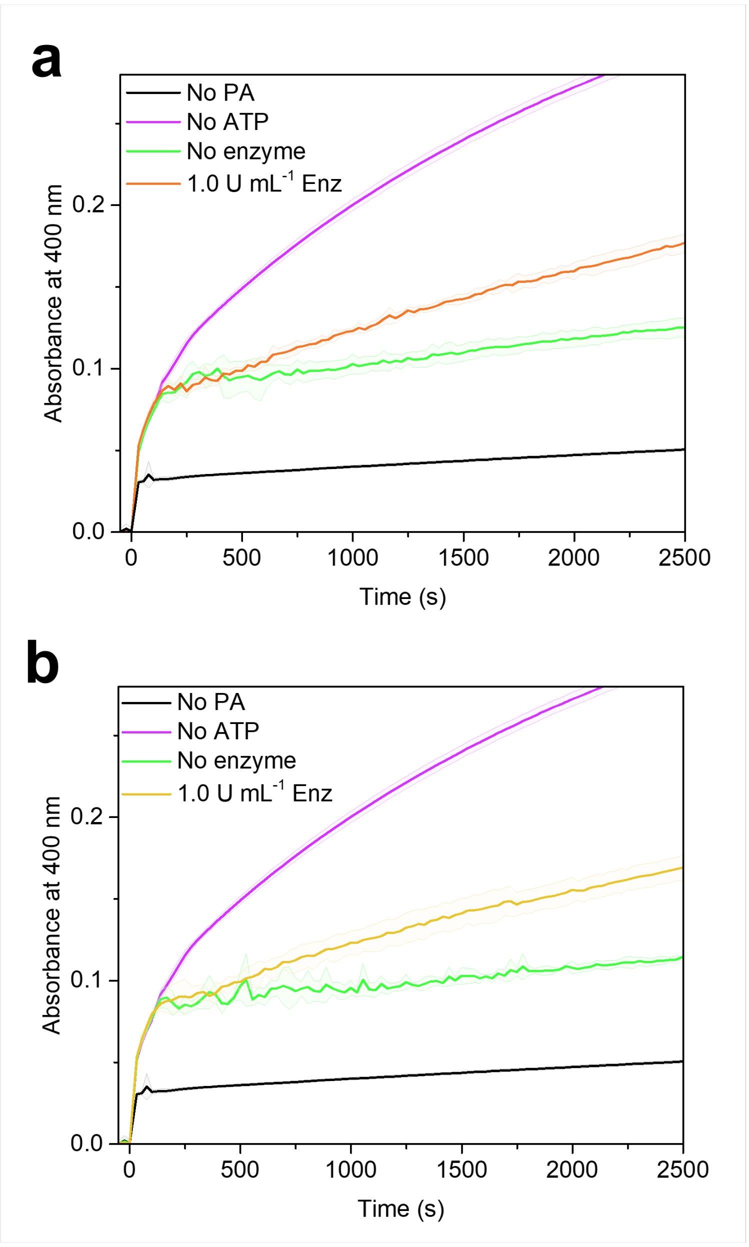


**Figure S14.** Kinetic experiments to prove the transient downregulation of PA reactivity by adding *p*-NPA (500 µM) at 0 s and (a) 60 µM or (b) 90 µM ATP after 100 s, in the presence of potato apyrase (1.0 U $mL^{-1}$) and PA (60 µM). In both cases, after a short inhibition time, the formation of *p*-NP starts to rise again. Experimental conditions: HEPES (10 mM, 7.2 pH), $CaCl_2$ (1.0 mM), 5% DMF to dissolve *p*-NPA, T = 30 °C, $\lambda_{abs.}$ = 400 nm. All the error bars denote standard deviation based on quadruplicate measurements.

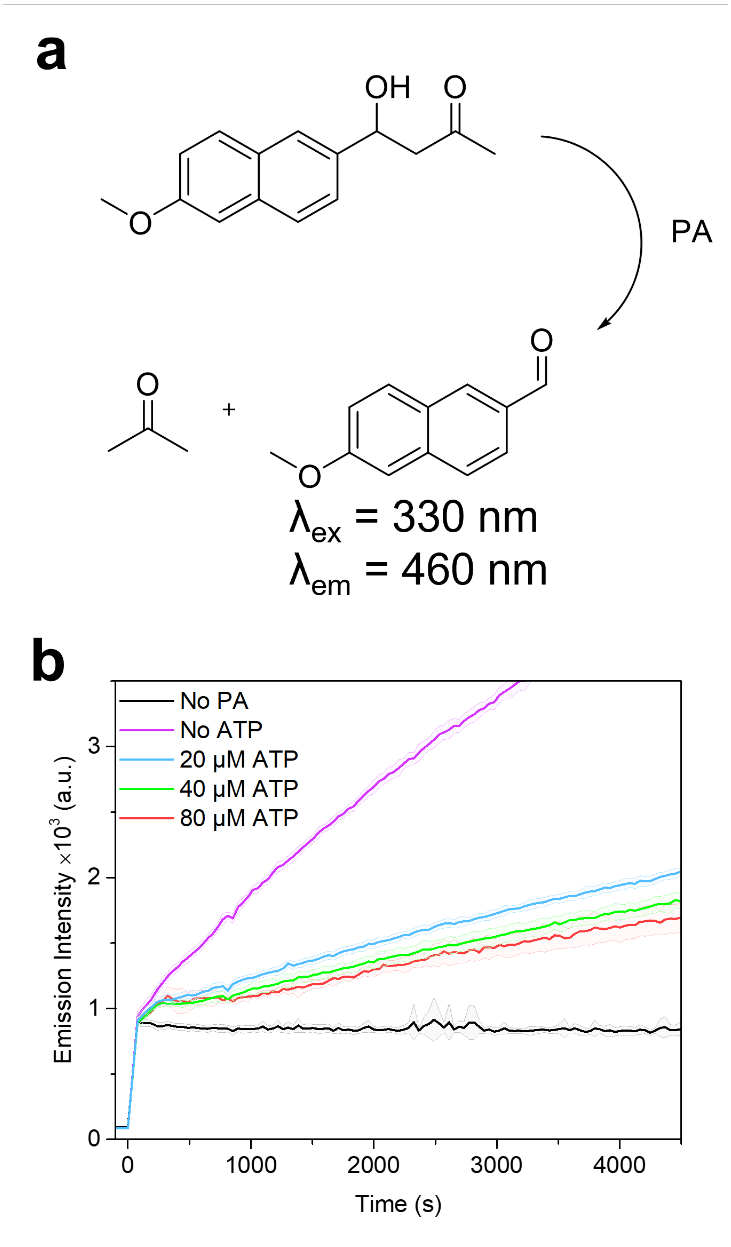


**Figure S15.** Kinetic experiments using 6-methoxy-2-naphthaldehyde as fluorescent product ($\lambda_{ex.}$ = 330 nm / $\lambda_{em.}$ = 460 nm) to study the catalytic activity regulation of PA. a) This probe is produced from the model retro-aldol reaction of methodol, catalyzed by PA. b) Fluorescence experiments where *p*-NPA (500 µM) was added at 0 s and ATP after 190 s, in the presence of potato apyrase (1.0 U $mL^{-1}$) and PA (40 µM). A short inhibition time of the retro-aldol reaction is observed. After that, fluorescence starts to increase again depending on the amount of ATP added, thus proving that ATP functions as a transient regulatory element for the downregulation of PA catalytic activity. Experimental conditions: $C_{16}V_3A_3K_3$ (40 µM), HEPES (10 mM, 7.2 pH), $CaCl_2$ (1.0 mM), 5% DMF to dissolve methodol, T = 30 °C.

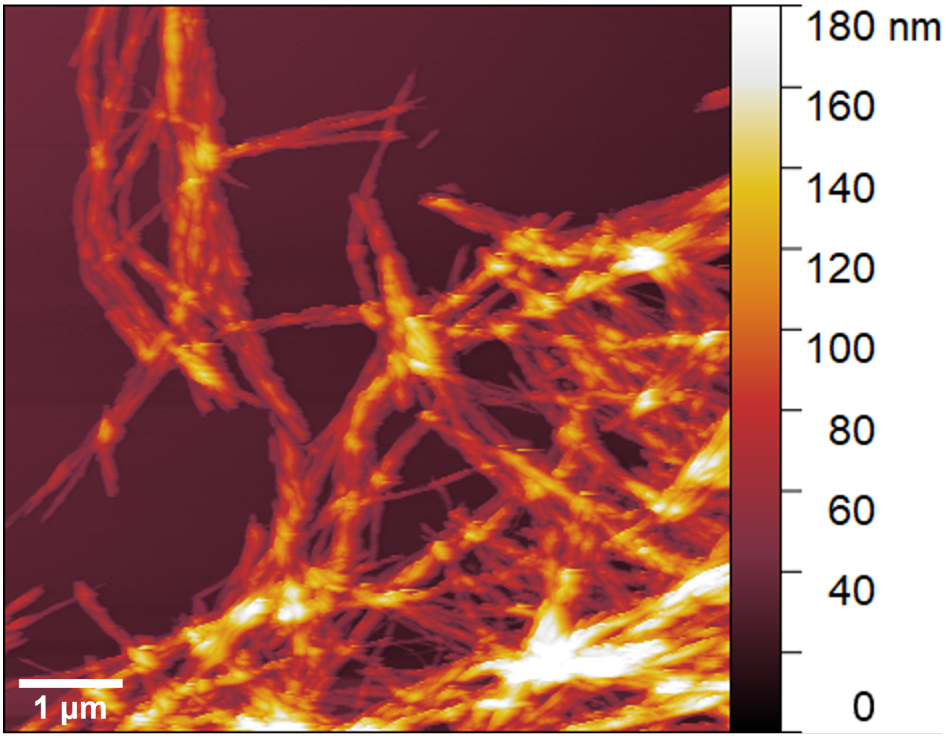


**Figure S16.** AFM images of annealed PA in the presence of ATP (60 µM) and ATPase (10 U mL$^{-1}$). After shaking the solution for 1 h, the imaged sample shows no reconversion to the initial thermodynamically favored PA nanofibers. Experimental conditions: $C_{16}V_3A_3K_3$ (100 µM), HEPES (10 mM, 7.2 pH), $CaCl_2$ (1.0 mM).

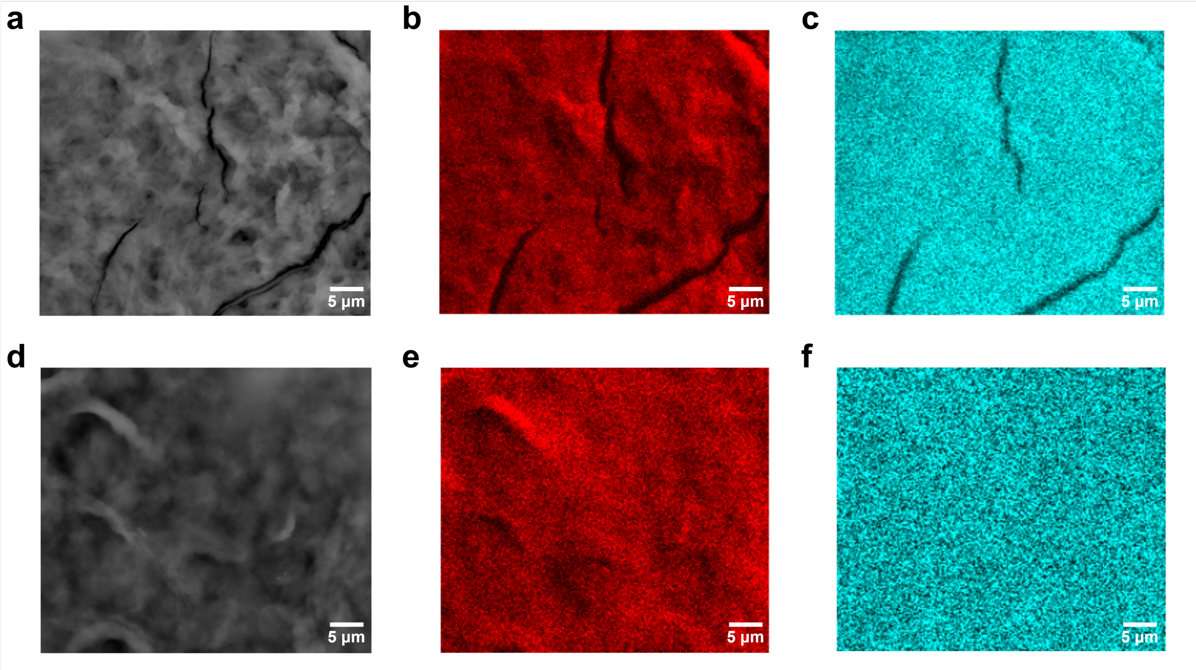


**Figure S17.** SEM-EDX analysis of samples: a,b,c) annealed PA in presence of ATP (1.0 mM) and d,e,f) annealed PA in presence of ATP (1.0 mM) and ATPase (10 U mL$^{-1}$). The red maps represent the content of carbon related to the peptide (b,e), while the blue maps the content of phosphorous related to ATP (c,f). Experimental conditions: $C_{16}V_3A_3K_3$ (1.0 mM), HEPES (10 mM, 7.2 pH), $CaCl_2$ (1.0 mM).

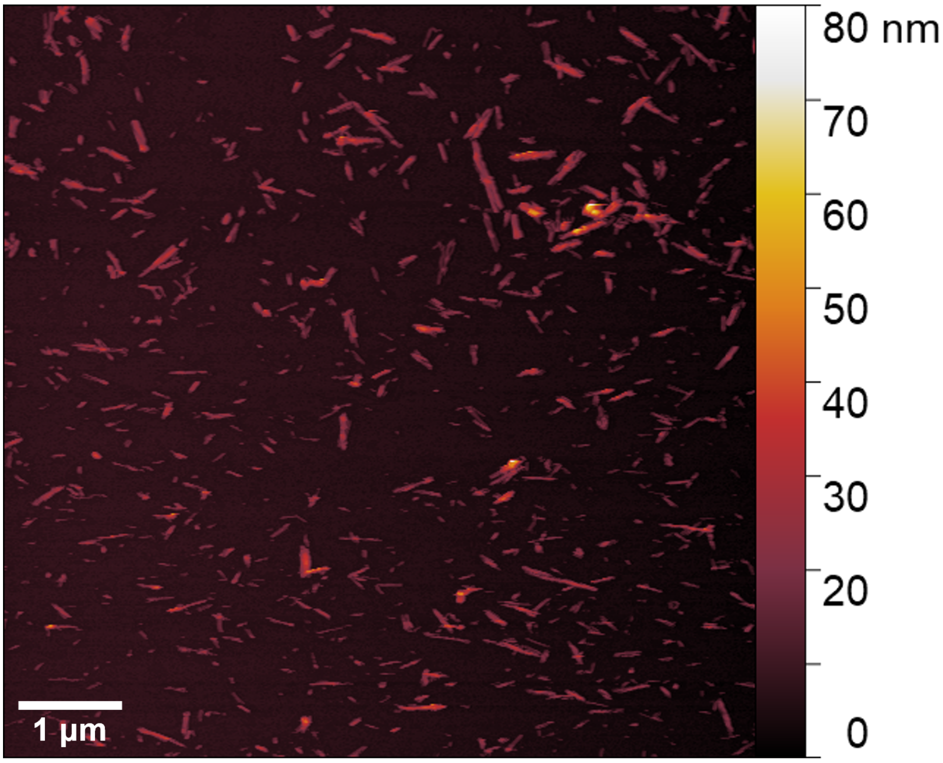


**Figure S18.** AFM images of annealed PA in the presence of ATP (60 μM) and ATPase (10 U $mL^{-1}$). After shaking the solution for 1 h and then sonicated (for 2 mins), the PA bundles broke into smaller fragments, leading to obtaining the morphology before thermal annealing. Experimental conditions: $C_{16}V_3A_3K_3$ (100 μM), HEPES (10 mM, 7.2 pH), $CaCl_2$ (1.0 mM).

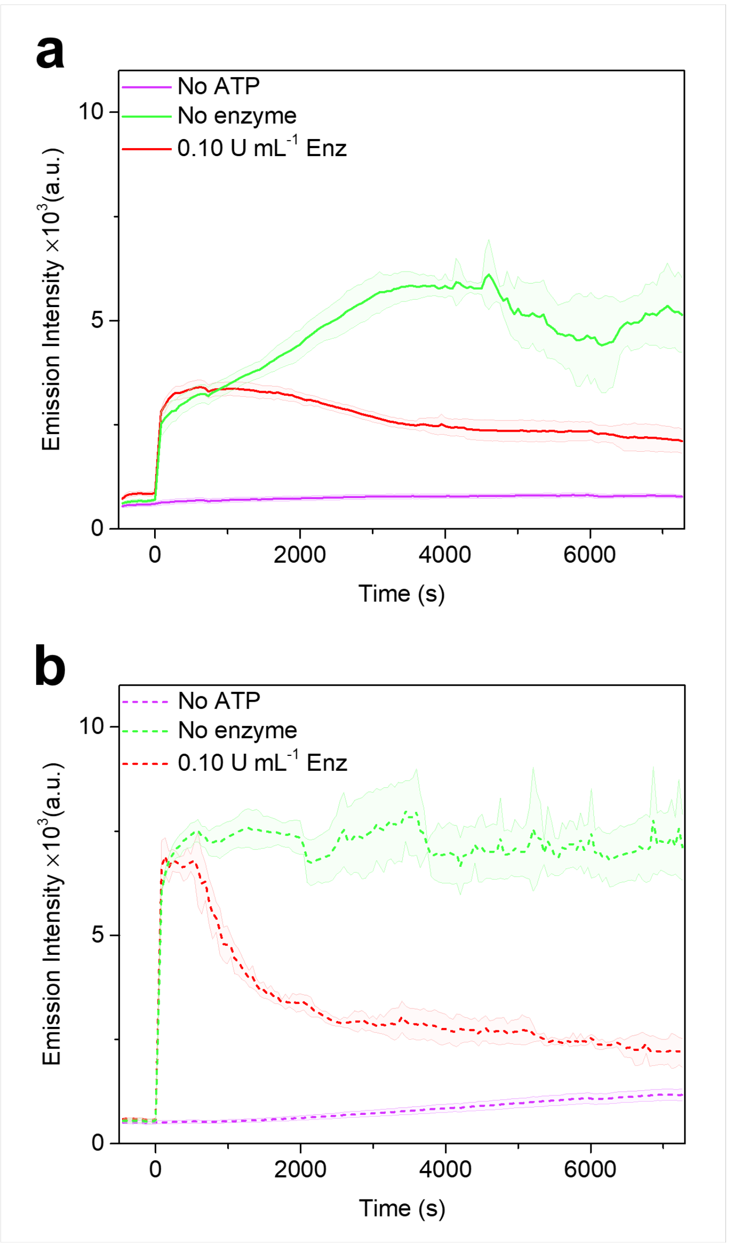


**Figure S19.** Kinetic fluorescence experiments with ThT (10 µM) using a) annealed PA nanofibers and b) polydisperse freshly dissolved PA nanofibers. For each sample, ATP (100 µM) was added at 0 s in the presence or absence of potato apyrase (0.10 U mL$^{-1}$). Experimental conditions: $C_{16}V_3A_3K_3$ (100 µM), HEPES (10 mM, 7.2 pH), $CaCl_2$ (1.0 mM), T = 30 °C, ThT 10 µM ($\lambda_{ex.}$ = 440 nm / $\lambda_{em.}$ = 483 nm). All the error bars denote standard deviation based on quadruplicate measurements.

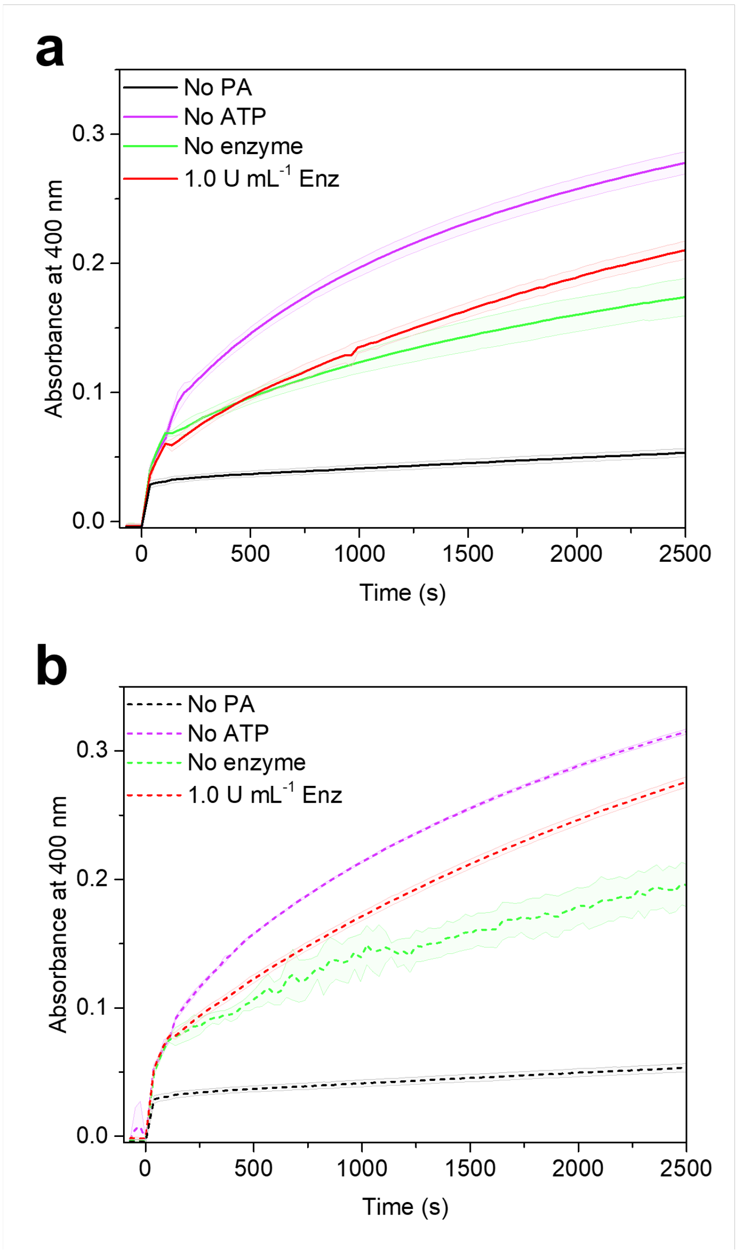


**Figure S20.** Kinetic UV-Vis experiments following model deacetylation reaction of *p*-NPA using a) thermodynamically favored PA nanofibers and b) polydisperse freshly dissolved PA nanofibers. For each sample, *p*-NPA (500 µM) was added at 0 s and ATP (30 µM) after 110 s in the presence or absence of ATPase (1.0 U mL$^{-1}$). Experimental conditions: $C_{16}V_3A_3K_3$ (60 µM), HEPES (10 mM, 7.2 pH), $CaCl_2$ (1.0 mM), 5% DMF to dissolve *p*-NPA, T = 30 °C, $\lambda_{abs.}$ = 400 nm. All the error bars denote standard deviation based on quadruplicate measurements.

# References


[1] C. A. Schneider, W. S. Rasband, K. W. Eliceiri, *Nat. Methods* **2012**, *9*, 671.
[2] D. Nečas, P. Klapetek, *Open Phys.* **2012**, *10*, 181.
[3] J. Boekhoven, C. M. Rubert Pérez, S. Sur, A. Worthy, S. I. Stupp, *Angew. Chem. Int. Ed.* **2013**, *52*, 12077.
[4] B. List, R. A. Lerner, C. F. Barbas, *J. Am. Chem. Soc.* **2000**, *122*, 2395.
[5] E. Elhalem, B. N. Bailey, R. Docampo, I. Ujváry, S. H. Szajnman, J. B. Rodriguez, *J. Med. Chem.* **2002**, *45*, 3984.
[6] S. Freire, M. H. De Araujo, W. Al-Soufi, M. Novo, *Dyes Pigm.* **2014**, *110*, 97.
[7] A. I. Sulatskaya, I. M. Kuznetsova, M. V. Belousov, S. A. Bondarev, G. A. Zhouravleva, K. K. Turoverov, *PLOS ONE* **2016**, *11*, e0156314.
